# The effect of potassium on aluminous phase stability in the lower mantle

**DOI:** 10.1007/s00410-024-02129-w

**Published:** 2024-04-27

**Authors:** Elena-Marie Rogmann, Eleanor S. Jennings, Jennifer Ross, Nobuyoshi Miyajima, Michael J. Walter, Simon C. Kohn, Oliver T. Lord

**Affiliations:** 1 School of Earth Sciences, University of Bristol, Bristol, BS8 1RJ UK; 2 Bayerisches Geoinstitut, Universität Bayreuth, Bayreuth, Germany; 3 Present Address: School of Natural Sciences, Birkbeck, University of London, London, WC1E 7JL UK; 4 Present Address: Earth and Planets Laboratory, Carnegie Institution for Science, Washington, DC USA

**Keywords:** Aluminous phases, Lower mantle, New aluminous phase, Calcium-ferrite-type phase

## Abstract

The aluminous calcium-ferrite type phase (CF) and new aluminous phase (NAL) are thought to hold the excess alumina produced by the decomposition of garnet in MORB compositions in the lower mantle. The respective stabilities of CF and NAL in the nepheline-spinel binary (NaAlSiO_4_–MgAl_2_O_4_) are well established. However with the addition of further components the phase relations at lower mantle conditions remain unclear. Here we investigate a range of compositions around the nepheline apex of the nepheline-kalsilite-spinel compositional join (NaAlSiO_4_–KAlSiO_4_–MgAl_2_O_4_) at 28–78 GPa and 2000 K. Our experiments indicate that even small amounts of a kalsilite (KAlSiO_4_) component dramatically impact phase relations. We find NAL to be stable up to at least 71 GPa in potassium-bearing compositions. This demonstrates the stabilizing effect of potassium on NAL, because NAL is not observed at pressures above 48 GPa on the nepheline-spinel binary. We also observe a broadening of the CF stability field to incorporate larger amounts of potassium with increasing pressure. For pressures below 50 GPa only minor amounts ($<0.011(1)\frac{K}{K+Na+Mg}$) of potassium are soluble in CF, whereas at 68 GPa, we find a solubility in CF of at least $0.088(3)\frac{K}{K+Na+Mg}$. This indicates that CF and NAL are suitable hosts of the alkali content of MORB compositions at lower mantle conditions. For sedimentary compositions at lower mantle pressures, we expect K-Hollandite to be stable in addition to CF and NAL for pressures of 28–48 GPa, based on our simplified compositions.

**Supplementary Information:**

The online version contains supplementary material available at 10.1007/s00410-024-02129-w.

## Introduction

During the process of subduction, significant amounts of oceanic crust with a MORB composition, as well as potentially some sediments, are transported into the Earth’s lower mantle. Both of these components are significantly richer in alumina and alkalis than the peridotitic mantle. KLB-1, a composition often used to represent fertile peridotitic mantle, has an Al_2_O_3_-content of approximately 3.6 wt.%, a Na_2_O-content of about 0.30 wt.% and a K_2_O-content of 0.02 wt.% (Takahashi 1986; Herzberg et al. 1990; Davis et al. 2009). These values are in stark contrast to those obtained for average MORB compositions, for which the average Al_2_O_3_-content is about 14.7 wt.%, the average Na_2_O-content about 2.8 wt.% and the average K_2_O-content about 0.16 wt.% (Gale et al. 2013). Sediments make up only a small volume fraction of a subducting slab, which is reduced further due to processes including fluid expulsion and offscraping, during the early stages of subduction (Poli and Schmidt 2002). Evidence for sediment offscraping has been found in subduction associated metamorphic complexes (e.g. Isozaki et al. 1990; Kimura and Ludden 1995), as well as through seismic imaging (e.g. Moore et al. 1991; Ito et al. 2009; Kimura et al. 2010). However, carbon and nitrogen isotopic evidence from eclogitic diamonds suggests a sedimentary component survives to transition zone depths at minimum (Cartigny 2005). If the sedimentary layer does remain attached to the slab and gets subducted into the deep Earth, it could contribute significant amounts of both alumina and alkali elements locally, as certain sediment facies can hold up to about 20 wt.% Al_2_O_3_ (Irifune et al. 1994), though more typical values are around 12 wt.% (Plank and Langmuir 1998). Sedimentary Na_2_O content are about 2.4 wt.% (Plank and Langmuir 1998), but can be as high as 5.8 wt.% (Baturin 2017) and K_2_O-content typically are around 2 wt.% (Plank and Langmuir 1998), but can reach up to 4 wt.% (Baturin 2017).

In the Earth’s peridotitic lower mantle the main phase assemblage is well established to be bridgmanite, ferropericlase and Ca-perovskite (e.g. Irifune 1994; Lee et al. 2004; Ono et al. 2005). The lower alumina and alkali contents of peridotitic compositions can be fully dissolved in this phase assemblage (e.g. Wang and Takahashi 2000; Wood 2000). However, in the basaltic part of a subducting slab at lower mantle conditions, additional phases are required to hold the excess Al_2_O_3_, Na_2_O and K_2_O (e.g. Ono et al. 2001; Hirose and Fei 2002). In the uppermost lower mantle majoritic garnet can still hold the excess Al and Na (Irifune 1987; Miyajima et al. 1999; Hirose and Fei 2002). Outside the stability field of garnet, NAL (new aluminous phase) and CF (calcium-ferrite type phase) take up the excess alumina (Miyajima et al. 1999). They are expected to constitute up to 20 volume % of MORB at lower mantle conditions (Walter et al. 2011; Mookherjee et al. 2012).

The calcium-ferrite type phase is orthorhombic in the *Pbnm* space group (Yamada et al. 1983), while NAL is hexagonal in the *P6*_3_/*m* space group (Miyajima et al. 2001). The CF general formula *AB*_2_*O*_4_ contains mono- and divalent cations on the A-site (Reid and Ringwood 1969; Liu 1977; Irifune et al. 1991). NAL has the general formula *AX*_2_*Y*_6_*O*_12_ and can also contain mono- or divalent cations on the A-site (Miyajima et al. 1999; Gasparik et al. 2000; Miyajima et al. 2001). Both phases are aluminous and due to their large cation sites, they can potentially hold a considerable fraction of the alkali budget of the lower mantle. CF was found to preferentially host Na_2_O over NAL, while being virtually K-free, suggesting that the K^+^-cation is too large to enter the CF structure (Miyajima et al. 2001). NAL, however, has been shown to accommodate potassium in its A-site (Miyajima et al. 1999; Gasparik et al. 2000; Miyajima et al. 2001; Hirose and Fei 2002; Kojitani et al. 2011). Significant potassium has been suggested to stabilise NAL to higher pressures than is observed for K-free or K-poor systems (Miyajima et al. 2001). In K-poor systems NAL is expected to be stable to approximately 20–50 GPa, though the exact transformation pressure to CF is composition dependent (Ricolleau et al. 2008; Ono et al. 2009; Ricolleau et al. 2010; Imada et al. 2011; Kawai and Tsuchiya 2012). CF is expected to be stable up to at least 134 GPa in MORB compositions (Hirose et al. 2005) and, thus, throughout Earth’s lower mantle. The structure is adopted by a range of compositions at high pressure. These include nepheline (NaAlSiO_4_; Liu 1977) and spinel (MgAl_2_O_4_; Irifune et al. 1991), which are most commonly studied, but also CaAl_2_O_4_ (Reid and Ringwood 1969), among others (e.g. Reid and Ringwood 1969; Akimoto et al. 2006). In MORB compositions, CF is expected to have a complex chemical composition (e.g. Ishii et al. 2022a).

Previous studies on the stability fields of NAL and CF are typically based on simple chemical systems. Among those, the most commonly studied is the NaAlSiO_4_-MgAl_2_O_4_ binary (Guignot and Andrault 2004; Ono et al. 2009; Imada et al. 2011). Some studies were also conducted along the CaAl_2_O_4_-MgAl_2_O_4_ binary (Akaogi et al. 1999; Ono et al. 2009; Mookherjee et al. 2012; Kawai and Tsuchiya 2012; Kimura et al. 2021), and the KAlSiO_4_-NaAlSiO_4_ binary (Mookherjee et al. 2012). Also notable is the study by Kato et al. (2013), who investigated compositions along the KAlSiO_4_-NaAlSiO_4_-binary but with the addition of a constant amount of MgAl_2_O_4_. Recently, Ishii et al. (2023) studied compositions within the NaAlSiO_4_-MgAl_2_O_4_-Fe_3_O_4_ ternary, though did not focus on investigating phase stability. While CF and NAL were also observed in more complex compositions, stability fields were not determined (e.g. Irifune and Ringwood 1993; Gasparik et al. 2000; Miyajima et al. 2001; Ono et al. 2002; Vanpeteghem et al. 2003; Guignot and Andrault 2004; Perrillat et al. 2006; Sanehira et al. 2006; Ricolleau et al. 2010; Ishii et al. 2019). While there is some preceeding experimental work on samples holding some potassium (Gasparik et al. 2000; Miyajima et al. 2001; Ono et al. 2002; Vanpeteghem et al. 2003; Guignot and Andrault 2004; Ishii et al. 2019), the effect of potassium on phase stability for pressures and bulk compositions relevant to the mantle remains poorly known. Generally, it is well established that NAL can hold significant potassium at low pressures (e.g. Hirose and Fei 2002; Vanpeteghem et al. 2003; Guignot and Andrault 2004; Kato et al. 2013; Ishii et al. 2022a), while the opposite was found for CF (Miyajima et al. 2001; Hirose and Fei 2002), so far confirming the argument of Miyajima et al. (2001) that the K^+^ cation is too large to enter the CF structure. However, the direct effect of K-solubility on the relative stability of CF and NAL has so far only been studied by Kato et al. (2013). In their study they used a 1:2 molar mixture of nepheline and spinel and replaced various amounts of nepheline with kalsilite. With this they confirmed a stabilizing effect of K on NAL and suggest the phase might be stable up to pressures of the core-mantle boundary, if the composition exceeds a molar ratio of 0.35 $\frac{\textrm{K}}{\textrm{K}+\textrm{Na}}$.

Only one suite of diamonds has so far been reported to contain inclusions of CF and NAL on the basis of their chemical compositions, namely the sublithospheric diamonds found in the Juina region, Brazil (Walter et al. 2022). These inclusions are rich in elements that are not abundant in the peridotitic mantle. The isotopic composition of the diamonds, alongside other indicators, suggest that they formed at the interface between the peridotitic mantle and subducted oceanic crust at deep transition zone to uppermost lower mantle depths (Walter et al. 2011; Thomson et al. 2014; Zedgenizov et al. 2014; Thomson et al. 2016). The CF and NAL inclusions contain up to 6.4 wt.% K_2_O and the presence or absence of potassium was used as the main distinction between CF and NAL (Thomson et al. 2014). The discovery of these inclusions highlights the importance of studying the effect of a potassic component on the stability of the CF and NAL phases at lower mantle conditions in MORB compositions, which is still poorly understood. This study presents a new set of experiments which investigate the stabilities and composition of the NAL and CF phases over the pressure range 28–78 GPa, in order to better understand phase relations in subducting slabs and the origin of superdeep diamonds. We examine the effect of potassium on the well-understood NaAlSiO_4_–MgAl_2_O_4_ binary system by investigating a range of compositions within the poorly studied nepheline-spinel-kalsilite (KAlSiO_4_) system.

## Materials and methodology

### Starting material

**Fig. 1:**
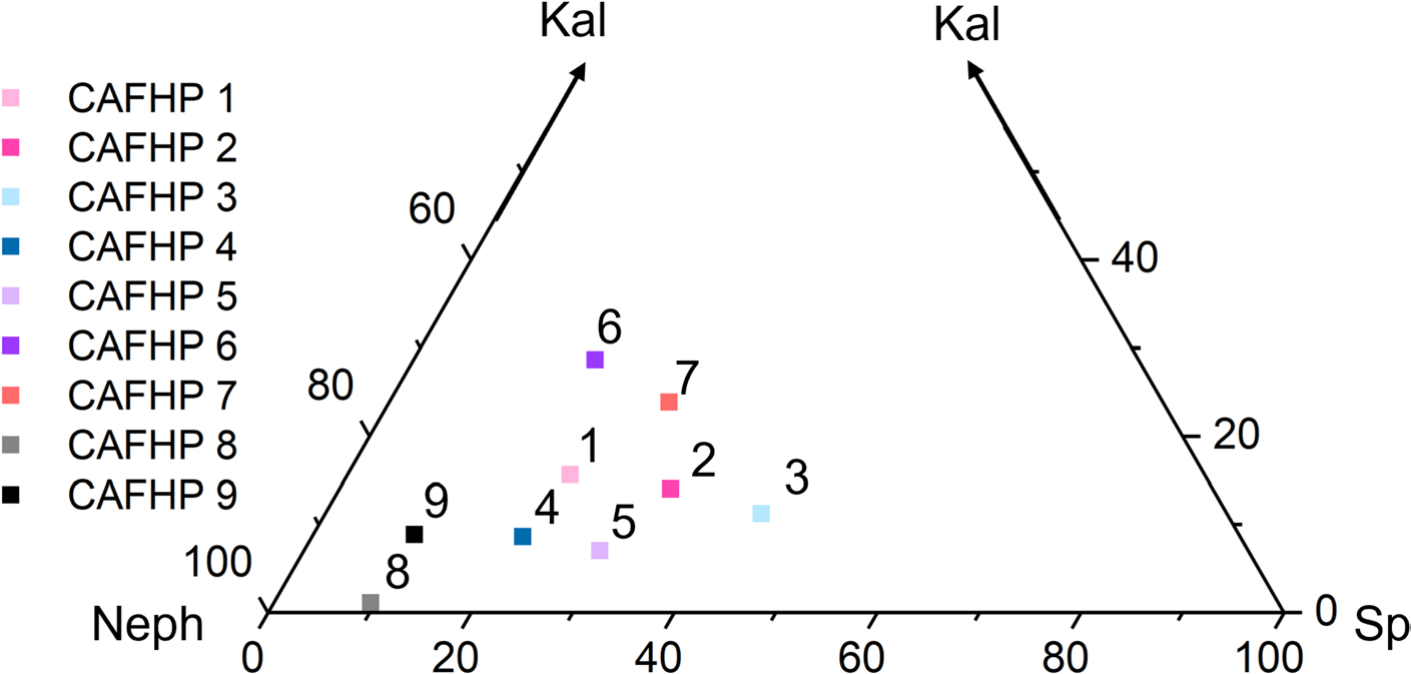
Projections of the CAFHP starting material compositions used in the present study onto the nepheline-spinel-kalsilite join. Kal = kalsilite; Neph = nepheline; Sp = spinel

Nine starting compositions were synthesised in the nepheline-kalsilite-spinel (NaAlSiO_4_, KAlSiO_4_, MgAl_2_O_4_) compositional system to examine phase relations around the nepheline apex. End-member nepheline and kalsilite were obtained by mixing high-purity oxides and carbonates (Al_2_O_3_, SiO_2_, K_2_CO_3_ and Na_2_CO_3_). Each powder was dried in a ceramic crucible at 600 ^∘^C and was, subsequently, reground. K_2_CO_3_ and Na_2_CO_3_ were decarbonated and all powders were annealed at 1000 ^∘^C in a platinum crucible overnight. The spinel composition was obtained by mixing Al_2_O_3_ and MgO, which was dried at 600 ^∘^C. All starting compositions were created as glasses, from mixtures of the synthetic end-members loaded into welded platinum capsules that had previously been annealed at 1000 ^∘^C. The ends were crimped closed and the capsules were initially held at 1000 ^∘^C for one hour. The capsules were then sealed by welding and placed in a vertical furnace at 1650 ^∘^C for 15 min in order to melt their contents. The capsules were then quenched in water. Breached capsules were rejected and repeated. Starting glasses were then crushed and finely ground with 10 wt.% Pt black powder, which acted as a laser-absorber. A chip of each of the glasses was retained and analysed by electron probe microanalyser at the University of Bristol with a 20 µm diameter, 10 nA beam accelerated at 15 kV. The resulting glasses are listed in Table 1 and plotted in Fig. 1. While minor spinel was identified in some glasses by X-ray diffraction (XRD), the absence of spinel peaks in the experimental run products indicates that it reacted out during heating of the subsequent experiments.

**Table 1:** Starting glass compositions, as determined by EPMA

Comp	*n*	Weight %						Mole %		
		Na_2_O	MgO	SiO_2_	K_2_O	Al_2_O_3_	Total	Ne	Ka	Sp
CAFHP1	29	13.8(8)	6(1)	31(1)	5.2(3)	43(2)	99.7(9)	63(4)	15.6(8)	22(4)
CAFHP2	21	11.7(7)	9.4(9)	27(1)	4.7(3)	47(1)	99.9(8)	53(3)	14.0(8)	33(3)
CAFHP3	30	10.0(7)	12(1)	23(2)	3.7(3)	51(2)	99.8(07)	46(3)	11.2(9)	43(4)
CAFHP4	30	15.6(8)	6.0(9)	33(1)	2.9(2)	43(1)	100.0(7)	71(4)	8.6(4)	21(3)
CAFHP5	31	14.0(5)	8.3(7)	29(1)	2.3(1)	45.9(9)	99.7(7)	64(2)	7.0(3)	29(2)
CAFHP6	31	11.5(4)	5.0(5)	32.2(7)	9.3(2)	41(1)	99.3(7)	53(2)	28.6(6)	18(2)
CAFHP7	30	10.5(7)	8(1)	29(1)	7.8(4)	45(2)	99.8(8)	49(3)	24(1)	28(3)
CAFHP8	30	20.0(5)	2.80(6)	38.3(5)	0.36(3)	38.6(4)	100.0(7)	89(2)	1.1(1)	9.6(2)
CAFHP9	30	17.5(4)	2.82(5)	37.7(5)	2.90(8)	39.3(5)	100.2(8)	81(2)	8.8(3)	10.0(2)

Number in brackets is the measurement uncertainty at one standard deviation. The projection into the three component system is normalised to 100

*n* number of measurements, *Comp.* Composition, *Ne* Nepheline, *Ka* Kalisilite, *Sp* Spinel

### Experimental setup

High pressure and temperature synthesis experiments were conducted in ‘Princeton-type’ diamond anvil cells (DAC), using anvils with 250 or 300 µm diameter culets. The cells were prepared by pre-indenting rhenium gaskets to a thickness of approximately 50 µm and laser-drilling a 50–100 µm diameter sample chamber within the indented gasket. The sample chambers were tightly packed with the starting material and some ruby powder at the edge of the sample chamber, between the starting material and the anvil. No pressure transmitting medium was used. Pressure was determined both before and after heating by the shift of the R1 ruby fluorescence peak, as calibrated by Mao et al. (1986). The post-experimental measurement was taken to represent the syn-experimental pressure without correcting for thermal pressure, which is typically around 10 % or less at these temperatures (Heinz 1990).

High temperatures were achieved using the double-sided laser heating and temperature measurement system installed at the School of Earth Sciences, University of Bristol and detailed in Lord et al. (2009). The two 100 W Yb-doped fibre lasers (SPI Red R4) with a wavelength of 1070 nm and a Gaussian energy distribution were focused down to a diameter of 40–50 µm. This beam diameter covered the bulk of the sample chamber, which typically had a 40–60 µm diameter after its initial collapse upon compression. The temperature was increased to the target and held constant for 15±2 min, at which point the sample was rapidly quenched by shutting off power to the lasers. Temperature was measured using standard spectroradiometric techniques (Walter and Koga 2004; Lord et al. 2009). Briefly, the incandescent light from the heated spot on both sides of the sample is focused on the entrance slit of an imaging spectrometer and is dispersed across a 2D CCD camera such that each row captures the spectrum from a point along a transect across the heated spot. Each of these spectra is then normalised to the spectral radiance of a NIST-calibrated W-lamp measured at the sample position and then fitted to the Wien approximation to the Planck function. This returns a 1D temperature profile across the experiment with a spatial resolution of around 3 µm. Analytical precision in temperature is typically 2–4 K, although experimental temperatures have far higher uncertainties (e.g. $\approx$ 150 K) arising from axial and radial thermal gradients, enhanced by the imperfect dissemination of the laser-absorbing Pt particles and temperature fluctuations during the experiment. Reported temperature uncertainties are the standard deviation of the maximum temperature over the experiment duration, averaged between the two sides.

Thirty eight experiments were completed at pressures ranging from 28–78 GPa and $\approx$ 2000 K. Experiments that heated poorly or gave large pressure or temperature errors (>150 ^∘^C) were omitted. We did not investigate a potential effect of temperature on the phase relations and used 2000 K as it is a commonly used estimate for temperatures in subducting slabs in the uppermost lower mantle (e.g. Hirose et al. 2005; Litasov and Ohtani 2005; Ishii et al. 2022a).

### Analytical methods

#### Synchrotron X-ray diffraction

Pressure- and temperature-quenched samples were analysed by angle dispersive powder X-ray diffraction, during two separate analytical sessions at Beamline I15, Diamond Light Source, UK. The monochromatic X-ray beams had wavelengths of 0.4428 Å or 0.4332 Å. The sample-to-detector distance was calibrated to a precision of ±10 µm using a silicon standard. In both cases, beam diameters were 15–30 µm, diffracted X-rays were detected using a MAR345 imaging plate with an acquisition time of 300 s, and some samples were rotated by up to 5^∘^ to obtain improved grain statistics. Diffraction patterns were integrated using FIT2D (Hammersley et al. 1995) and Dioptas (Prescher and Prakapenka 2015). Phases were identified using Macdiff and PDIndexer (Seto et al. 2010). Full-profile fitting was performed on selected patterns using GSAS-II (Toby and Von Dreele 2013).

#### FE-EPMA

Experimental run products were prepared for field emission gun electron probe microanalysis (FE-EPMA) by mounting in one inch epoxy disks and grinding until the sample surface was exposed. The surface was then polished with 1 µm diamond suspension and was carbon coated. FE-EPMA analysis was carried out at the University of Bristol using a JEOL JXA8530F Hyperprobe with an anorthoclase standard. Point analyses were performed with a 10 kV, 2 nA beam rastered over an area of 1 µm^2^. At each location, peak intensity was measured twenty times for one second per measurement. Using the same procedure, the background intensity for each area was measured above and below the peak. This procedure was adopted in order to minimise beam damage and loss of alkali elements. The initial K and Na contents were determined by extrapolating measured values to the zero-time concentrations. Uncertainties on bulk composition measurements are given as standard deviations of repeated analyses. Due to small crystal sizes in the run products, phases could not be measured individually by FE-EPMA. However, FE-EPMA data for experiments that had been shown by diffraction to contain only CF and NAL, were used to determine the orientation of compositional tie lines between the two phases. This approach assumes that each analysis represents a physical mixture of the two phases, but in variable proportions from analysis to analysis. In run products that also exhibited other phases, this approach is not viable. Within the measurement data on the binary compositions, data was discarded if their microprobe totals were lower than 95 wt.% or if their recalculated mineral composition was non-stoichiometric for lower mantle aluminous phases. For a total of four oxygens, the ideal composition here would be three cations. We discarded data which returned less than 2.85 or more than 3.15 cations per formula unit. The discarded data points were interpreted to be a result of an uneven sample surface, as sample polishing was challenging, due to the small grain size. Additionally, elemental maps were created with a 10 kV, 10 nA beam, with a 0.1 µm step size and a 20 ms dwell time.

#### FIB preparation

A focused ion beam (FIB) at the Universität Bayreuth was used to cut TEM lamellae from experiments CAFHP6-4 and CAFHP2-3. The lamellae were cut in the direction of compression, perpendicular to the diamond culet with a Ga^+^-beam by first cutting two parallel trenches and progressively thinning the central part with a successively lower current beam. The lamellae were extracted and welded to a Cu-grid with Pt and were thinned to around 100–200 nm, using a progressively lower energy beam to reduce beam damage and remove Gallium surface contamination. Beam conditions ranged from 30 kV and 50 nA to 2 kV and 27 pA for the final thinning.

#### TEM analysis

The two lamellae were then examined by transmission electron microscopy (TEM), using the FEI Titan G2 80-200 S/TEM at the Bayerisches Geoinstitut, Universität Bayreuth. The analyses were conducted to determine the compositions of the various phases and used both conventional TEM and STEM modes. An electron beam accelerated at 200 kV was generated by an extreme brightness field emission gun (X-FEG) Schottky electron source. In STEM mode, the high angle annular dark field (HAADF) detector was used to acquire signal. For energy dispersive X-ray spectroscopy (EDX) measurements, a windowless Super X-EDS detector with 4 silicon-drift detectors (SDDs) was used. Selective area electron diffraction (SAED) patterns were taken to confirm phase identity and EDX spot analyses were used to measure the composition of the identified phases. STEM mapping was used to determine average phase compositions over broader areas. Beam damage was minimised relative to other measurement techniques by rastering the beam continuously during the measurement. Rastered areas covered a few square micrometers for each experiment. Average phase compositions were taken from a rectangular region covering most of the grain. A standardless analysis technique was applied, whereby element concentrations were determined through calibrated and interpolated k-factors for thin TEM foils. The requirement for stoichiometric oxygen allowed the sample thickness to be estimated for the absorption correction (Van Cappellen and Doukhan 1994).

## Results

### Phase relations and textural observations

Thirty-eight experiments were completed at pressures from 28 to 78 GPa and around 2000 K, of which 11 were omitted due to large pressure and/or temperature uncertainties. The run conditions and resulting phase assemblages, as determined by powder-XRD, are given in Table 2 (1D integrated diffraction patterns are provided in the digital supplementary materials on the Bristol data repository). Phase assemblages, starting compositions and phase compositions are graphically represented in ternary diagrams (Figs. 4, 6).

#### Phase assemblages

CF was found to be a stable phase in all experimental run products. NAL was found to be an additional stable phase for experiments with kalsilite-rich starting materials (>11.2 mole%). We observed NAL up to pressures of 71 GPa, always co-occurring with CF.

Additional phases aside from CF and NAL are observed in many of the experiments. Most frequently these are K-Hollandite (KAlSi_3_O_8_) and $\delta$-phase (AlOOH), usually, in combination (Table 2). Together they approximate the stoichiometry of the kalsilite component, suggesting that its solubility in CF and NAL has been exceeded. We argue that the presence and coexistence of K-Hollandite with $\delta$-phase is a result of the decomposition of kalsilite at high pressures, which is consistent with the findings of Liu (1978b). $\delta$-phase is also observed in the absence of K-Hollandite, though it often exhibits less intense XRD peaks. In those cases an alternative explanation is a partial hydration of the ruby pressure marker (Al_2_O_3_). $\delta$-phase is generally expected to be a stable phase at the pressure and temperature conditions applied here (Ohira et al. 2014; Duan et al. 2018; Ishii et al. 2022b). At pressures below 40 GPa, however, partial (Duan et al. 2018) or total dehydration (Ohira et al. 2014; Ishii et al. 2022b) of $\delta$-phase is expected for the temperatures applied here. Temperature gradients, leading to lower temperatures at the boundary of the laser heated region, are a likely explanation for the observation of $\delta$-phase in experiments performed at temperatures nominally above the dehydration reaction boundary. Notably, corundum is exclusively observed in some, though not all, of the experiments conducted below 40 GPa (Table 2), where partial dehydration is expected (Duan et al. 2018). Whether the presence of corundum represents the ruby pressure marker or is a product of the dehydration of $\delta$-phase cannot be definitively stated. One experiment exhibits K-Hollandite without the $\delta$-phase. The presence of corundum in that experiment suggests that it could also represent a breakdown product of the kalsilite component and might be present, rather than $\delta$-phase, due to its dehydration at low pressures (Ohira et al. 2014; Duan et al. 2018; Ishii et al. 2022b). The stoichiometry of the $\delta$-phase as measured by TEM is approximately (Al_0.85_Si_0.1_Mg_0.05_)OOH. This concentration of Si and Mg is consistent with reported compositions of high-pressure $\delta$-AlOOH (e.g. Suzuki et al. 2000; Komatsu et al. 2011). Based on Liu (1978b) pure kalsilite would be expected to disproportionate to K-Hollandite with KAlO_2_ for pressures up to at least 30 GPa. For the experiments conducted here we do not observe a KAlO_2_ phase in the XRD patterns, nor do we observe high enough K-contents in the EPMA or TEM data to verify such a phase. Alternatively, if Al_2_O_3_ and K_2_O are stable instead of KAlO_2_, the K_2_O would not survive at room pressure and temperature because of reaction with water in the atmosphere, and only the Al_2_O_3_ (or $\delta$-phase in the case of samples with some water present) would be detectable by XRD. In some experiments we also find evidence of periclase (MgO), stishovite (a high pressure polymorph of SiO_2_) and hydrous phase D, reported to have an ideal stoichiometry of MgSi_2_O_6_H_2_ (Frost and Fei 1998). Some experiments also exhibit corundum peaks, which are likely due to the ruby that was used as a pressure indicator. The formation of hydrous phases indicates the presence of residual moisture in the starting material. The presence of a small hydrous component is supported by the EPMA totals, which are frequently lower than 100 wt.% (supplementary information, Tables S1–S2).

**Table 2:** Experimental conditions and resulting phase assemblages, as identified from XRD patterns

Comp	Exp	P (GPa)	T (K)	1$\sigma$(T) (K)	Observed phases
CAFHP1	1-1	31.2	1989	104	CF+NAL+K-Holl+$\delta$
	1-2	50	1994	67	CF+NAL
	1-3	65.1	2084	75	CF+NAL
	1-4	39.3	1916	114	CF+NAL+K-Holl+$\delta$
CAFHP2	2-1	29.8	2012	44	CF+NAL+K-Holl+Per+(Cor)
	2-3	56.1	2052	59	CF+NAL+$\delta$
	2-4	47.2	1992	54	CF+NAL+K-Holl+$\delta$
CAFHP3	3-1	32.7	1979	50	CF+NAL+K-Holl+$\delta$+D+Per(+Cor)
	3-2	52.5	2015	59	CF+NAL+$\delta$
CAFHP4	4-1	28.9	2032	50	CF+K-Holl+$\delta$+D+Per(+Cor)
	4-2	55.9	2033	151	CF+$\delta$
	4-3	77.7	2025	75	CF+$\delta$
CAFHP5	5-3	69	2007	41	CF+$\delta$
	5-4	40.7	1967	65	CF+K-Holl+$\delta$+Per
CAFHP6	6-3	42.8	1941	60	CF+NAL+K-Holl+$\delta$
	6-4	34.7	1995	87	CF+NAL+K-Holl+$\delta$
	6-6	44.7	1992	71	CF+NAL+K-Holl+$\delta$
CAFHP7	7-2	71	2035	64	CF+NAL
	7-3	37.7	1993	58	CF+NAL+K-Holl+$\delta$
	7-4	35.5	2004	91	CF+NAL+K-Holl+$\delta$
	7-5	53.5	2040	64	CF+NAL
CAFHP8	8-1	33.1	1984	48	CF+K-Holl+$\delta$
	8-2	53.6	2017	70	CF
CAFHP9	9-4	28.1	1945	50	CF+$\delta$+Stish(+Cor)
	9-5	40.9	1997	68	CF+K-Holl+$\delta$+Stish
	9-6	67.8	1992	69	CF
	9-7	48.9	1940	83	CF+K-Holl+$\delta$

P is the pressure obtained post-heating and does not include a thermal pressure correction. Estimated uncertainty in P is 1 GPa. Only experiments that presented homogenous heating of the sample material, without heating of the gasket, are included. The 1$\sigma$(T) uncertainty is one standard deviation in the temperature measurements from both sides of the DAC, with an additional 5 K fitting uncertainty. Corundum was interpreted to come from the ruby gauge. Comp. = Composition; Exp. = Experiment number, CF = calcium-ferrite type phase; NAL = new aluminous phase; K-Holl = K-Hollandite; $\delta$ = $\delta$-phase (AlOOH); Per = periclase; Cor = corundum; D: Hydrous phase D; Stish = stishovite

#### Experimental textures

FE-EPMA mapping of a polished surface perpendicular to the compression axis was performed on several experiments.

**Fig. 2:**
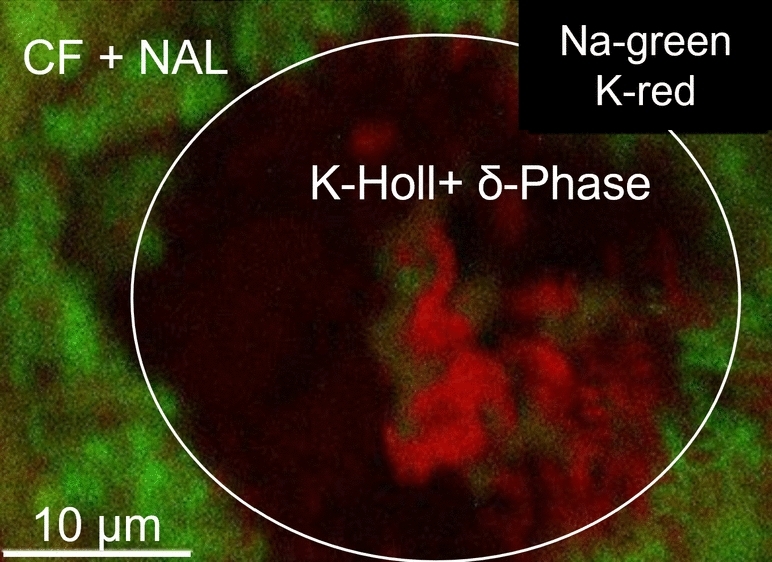
FE-EPMA Na and K element map for experiment 6-4, exhibiting strong chemical segregation with a potassium-rich central area and a sodium-rich outer ring. The inner section was found to be dominated by K-Hollandite and small proportions of $\delta$-phase. The outer ring is composed of a mixture of CF and NAL

**Fig. 3:**
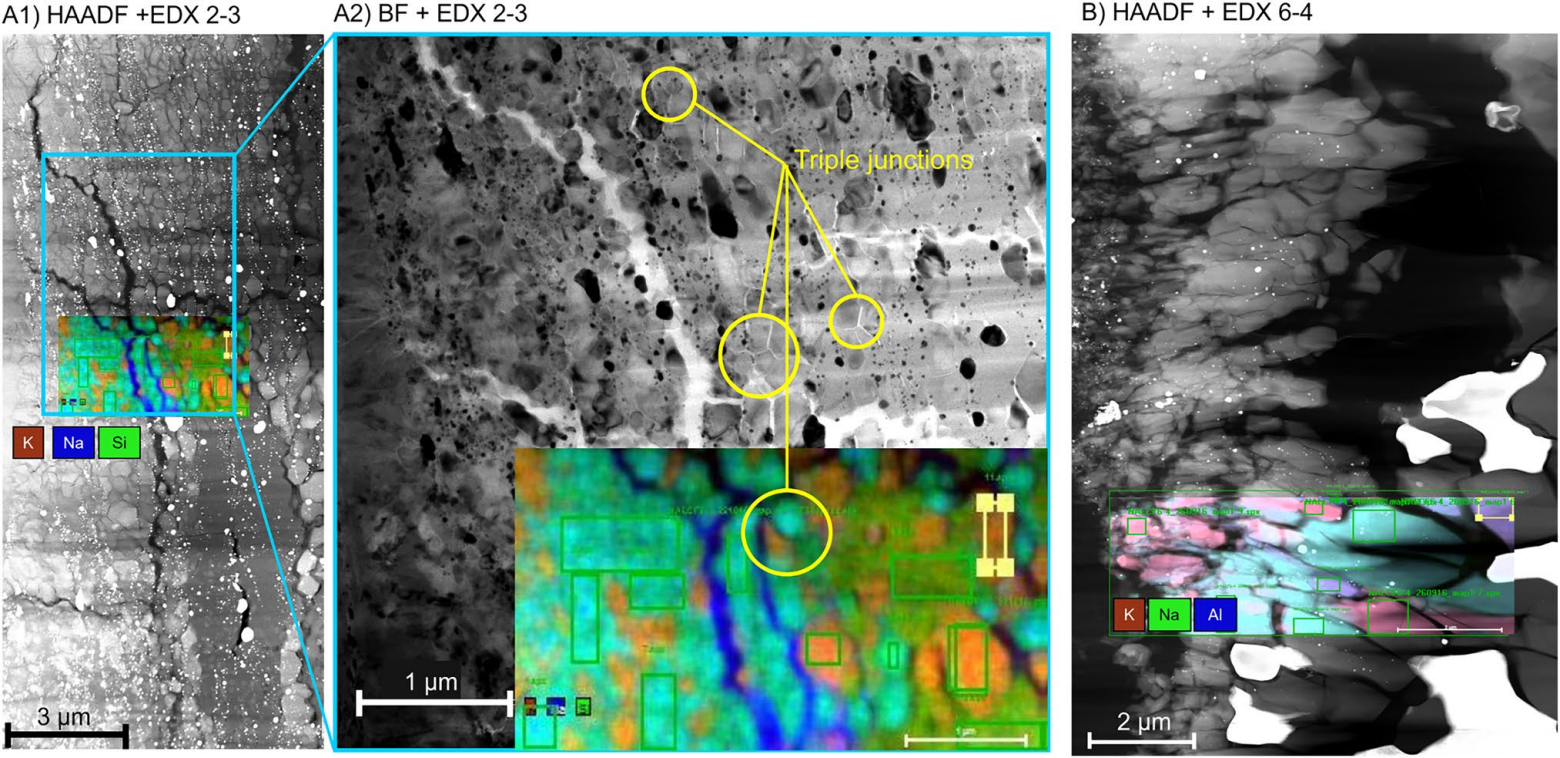
STEM images of the experiments CAFHP 2-3 (**A**) and CAFHP 6-4 (**B**). EDX images of the whole rastered area were overlain over bright field (BF) and high-angle annular dark-field (HAADF) STEM images; EDX: Energy dispersive X-ray spectroscopy. Multiple triple junctions can be observed in figure A2). In experiment 2-3 we observed the major phases CF, NAL. Orange colours indicate NAL grains, while turquoise grains are indicative of CF. For experiment 6-4, CF is of a similar turquoise colour, while NAL is purple and K-Hollandite is pink

In the unusual case of experiment CAFHP6-4 we observe chemical segregation (Fig. 2). This is indicated by a coarse-grained Al-rich central portion around a large globule of Platinum. Such textures are indicative of melting and subsequent amalgamation (Walter et al. 2015). The cooler rim around the hotspot is fine grained and rich in Na and K. This represents a subsolidus equilibrium assemblage, allowing us to use the associated compositional data to reconstruct phase relations on the nepheline-kalsilite-spinel compositional join, as the tie lines intersect a ’local bulk’ composition (Fig. 2). TEM imaging shows large, elongate crystals of up to 1 µm in the centre of the sample, which have been identified as K-Hollandite by SAED TEM electron diffraction (Fig. 3). The K-Hollandite was found to have a composition of (K_0.8_Na_0.1_Mg_0.1_)Al_1.3_Si_2.7_O_8_ (STEM measurement; O from stoichiometry) and coexists with $\delta$ phase. In contrast, the experiments at pressures exceeding 49 GPa exhibit a more homogeneous sample texture suggesting they were equilibrated below the solidus. This is confirmed by STEM imaging performed on a perpendicular section of CAFHP2-3, cut parallel to the compression axis, which shows a random mixture of sub-500 nm CF ± NAL crystals, and fine-grained, randomly distributed platinum grains. Occasional 120^∘^ triple junctions imply that equilibrium was attained (Fig. 3).

#### NAL and CF compositions

**Fig. 4:**
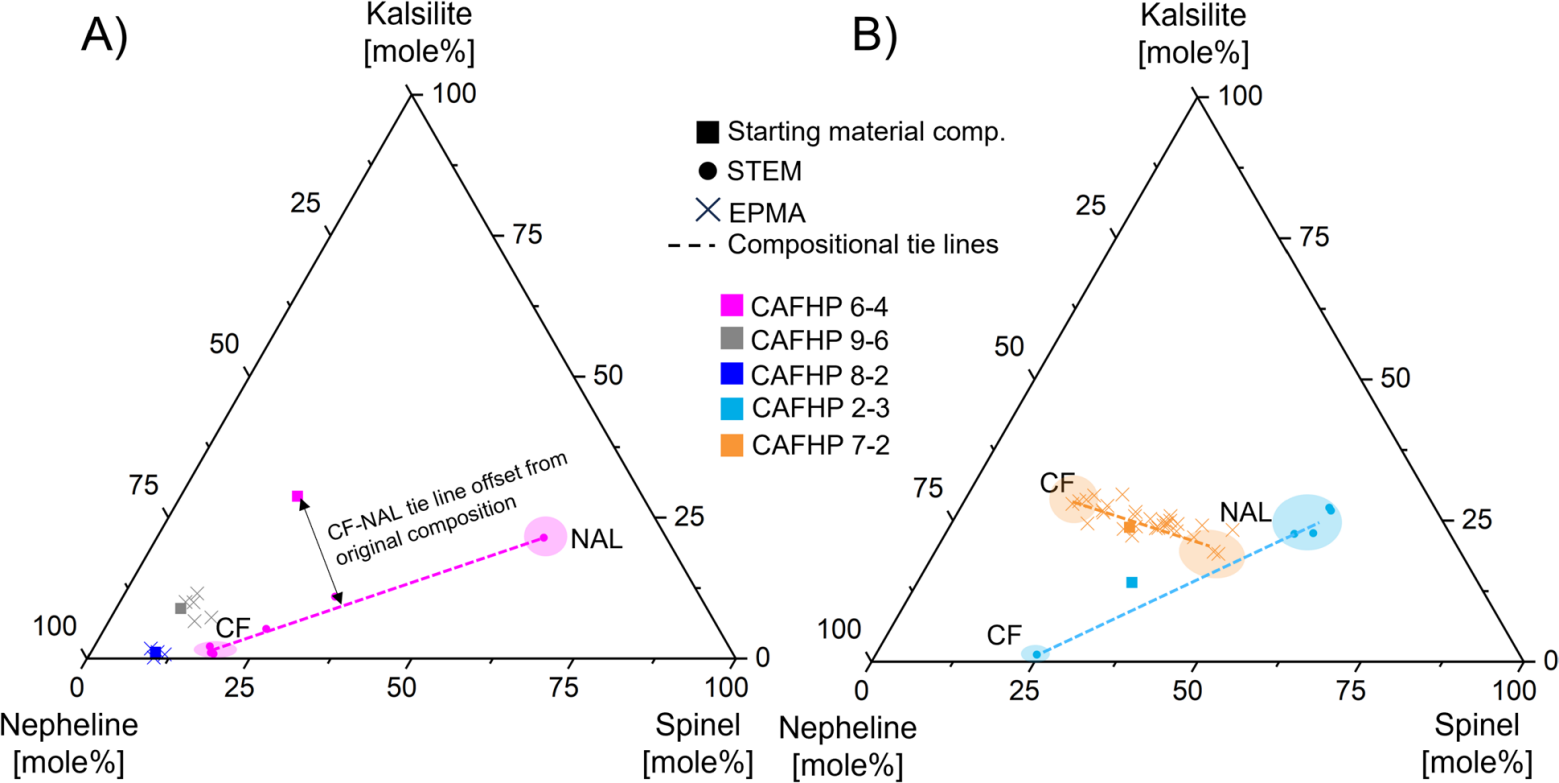
FEG-EPMA and EDX TEM compositional data on the nepheline-kalsilite-spinel compositional join for all experiments displaying statistically significant (R^2^ $\ge$ 0.5) chemical trends in the TEM or the microprobe data. Chemical trends are indicated as dashed lines and function as tie lines between the CF and NAL end-member compositions. **A** The microprobe data for CF single phase experiments 8-2 and 9-6 scatter around the starting material compositions, indicating the composition of the CF phase to be equivalent to the starting material compositions. The STEM CF-NAL compositional tie line of experiment 6-4 is offset to lower kalsilite contents relative to the starting material composition. The tie line intersects a local composition and the aluminous phases are in local equilibrium with that composition. The excess kalsilite component has formed an assemblage of K-Hollandite with $\delta$-phase. **B** The STEM tie line between CF and NAL compositions for experiment 2-3 (56 GPa) passes through the initial starting material composition, indicating chemical equilibrium was attained during the experiment. The microprobe data for experiment 7-2 (71 GPa) also form a tie line between CF and NAL compositions. The different angle is a result of broadening CF and NAL stability fields in the higher pressure region. The microprobe data for experiments that are not displayed here cluster around the starting material composition and do not exhibit statistically significant chemical trends

Phase compositions were determined by STEM analysis and by FE-EPMA (Fig. 4).

The bulk sample compositions as obtained by FE-EPMA through averaging of different point measurements returned compositions within uncertainty of the starting materials in all cases. Within the experimental run products, the grain size of the aluminous phases was determined to be in the range of hundreds of nanometers. This is smaller than the resolution of the FE-EPMA analysis (1 µm), leading to averages of multiple crystallites. FE-EPMA analyses of multi-phase assemblages exhibited a broader range of compositions than those for mono-phase run products. We observed CF to be the stable assemblage for the most kalsilite-poor compositions at lower pressures with compositions determined by FEG-EPMA clustered around that of the starting material (Fig. 4A). With increasing pressure, we observe more kalsilite-enriched compositions to also exhibit CF as the single stable phase (Table 2). This confirms the widening of the solid solution between Na-rich and K-rich CF with pressure. In contrast, the much higher resolution of STEM mapping allowed us to obtain the chemical compositions of individual phases. Two experiments were analysed by STEM: one containing two phases and one containing four phases. The measured CF and NAL phases are compositionally distinct from one another in both cases, with the NAL composition being richer in the spinel-component, and the CF composition being richer in the nepheline-component (Fig. 4). We note that the TEM tie line of CF and NAL compositions of experiment 6-4 is offset to a lower kalsilite-content than the starting material (Fig. 4). This offset is in agreement with our interpretation that K-Hollandite + $\delta$-phase represents the excess kalsilite component and that the phase relations are non-ternary in this part of the phase diagram, as discussed below. The chemical compositions of NAL and CF allow us to constrain the respective single phase stability fields in the ternary, with a wide two-phase field in between. The angle of the tie line indicates that in compositions with less than 70 mole % nepheline, potassium partitions preferentially into NAL, thereby stabilising it. In contrast, a composition close to the nepheline apex could crystallise CF, including larger amounts of a potassic component, as pressure increases. We also find that the compositional tie lines rotate towards the highest probed pressures (Fig. 4B), as a result of increasing K-solubility in CF.

### Phase proportions

**Table 3:** Overview of the Rietveld refinements of eight experiments

Exp	P (GPa)	Bulk composition (mole%)			Phase proportions				wR (%)
		Neph	Kal	Sp	CF	NAL	K-Holl	$\delta$	
1-2	50.0	62.5(3.5)	15.6(8)	22.0(4.0)	68.8(7.5)	31.2(7.5)	–	–	3.12
1-3	65.1	62.5(3.5)	15.6(8)	22.0(4.0)	69.2(7.5)	30.8(7.5)	–	–	2.51
2-3	56.1	53.3(3)	14.0(8)	32.7(3.1)	62.2(7.5)	35.6(7.5)	–	2.2(7.5)	2.07
3-2	52.5	45.8(3.3)	11.2(9)	43.0(3.8)	52.1(7.5)	30.8(7.5)	–	17.1(7.5)	2.39
5-3	69.0	63.8(2.4)	7.0(3)	29.2(2.3)	96.0(7.5)	–	–	4.0(7.5)	2.79
7-2	71.0	48.6(3.1)	23.8(1.1)	27.6(3.4)	42.2(7.5)	57.8(7.5)	–	–	2.74
7-5	53.5	48.6(3.1)	23.8(1.1)	27.6(3.4)	41.6(7.5)	58.4(7.5)	–	–	2.71
9-7	48.9	81.2(1.7)	8.8(3)	10.0(0.2)	76.9(7.5)	–	12.6(7.5)	10.5(7.5)	2.21

The weighted residuals indicate the quality of the refinement

*P* pressure, *wR* weighted residuals, *Neph* nepheline component, *Kal* kalsilite component, *Sp* spinel component, *CF* calcium-ferrite type phase, *NAL* new aluminous phase, *K-Holl* K-Hollandite

**Fig. 5:**
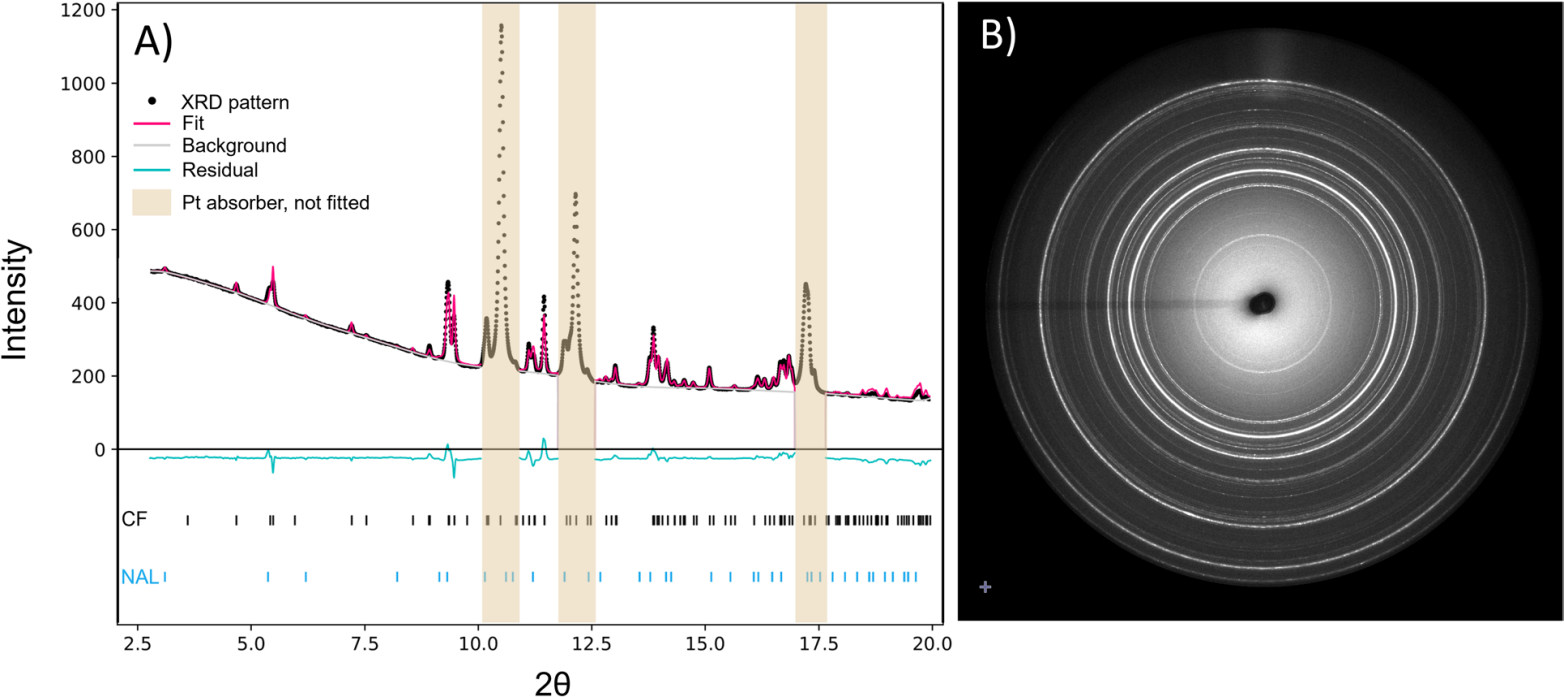
**A** An example of a Rietveld refinement and its residual for experiment 1-3 with CF and NAL. Regions dominated by Pt peaks, introduced as the absorber, were not included in the refinement and are shaded in yellow here. **B** The corresponding 2D diffraction image of experiment 1-3

For diffraction patterns of good quality and three or fewer phases, we conducted Rietveld refinements using GSAS II (Toby and Von Dreele 2013), to estimate relative phase proportions (Table 3). GSAS-II returned errors below 1% for the Rietveld refinements of the phase proportions. However, considering the nature of diamond anvil cell phase relation studies, with uniaxial stress components likely leading to preferred crystal orientations, we assume an uncertainty of ± 7.5% on the phase proportion of each phase. This is based upon the abundances of $\delta$-phase, which we expect to be similar for all experiments where present. With an error estimate of ± 7.5%, all abundances of the $\delta$-phase are within uncertainty of each other. In Fig. 5 we show an example of a Rietveld fit and its residual for experiment 1-3. Despite the significant uncertainty, we find that the abundance of CF increases with increasing nepheline content of the starting material and decreases with increasing kalsilite and spinel components. These relative abundances were used in the construction of the phase relations through application of the lever rule. The abundances of the $\delta$-phase and K-Hollandite are $\approx$ 10%. Lastly, we observe significant scatter in the abundance of the NAL component, with a slight trend to higher NAL proportions with increasing kalsilite contents (supplementary information, Figure S2).

**Fig. 6:**
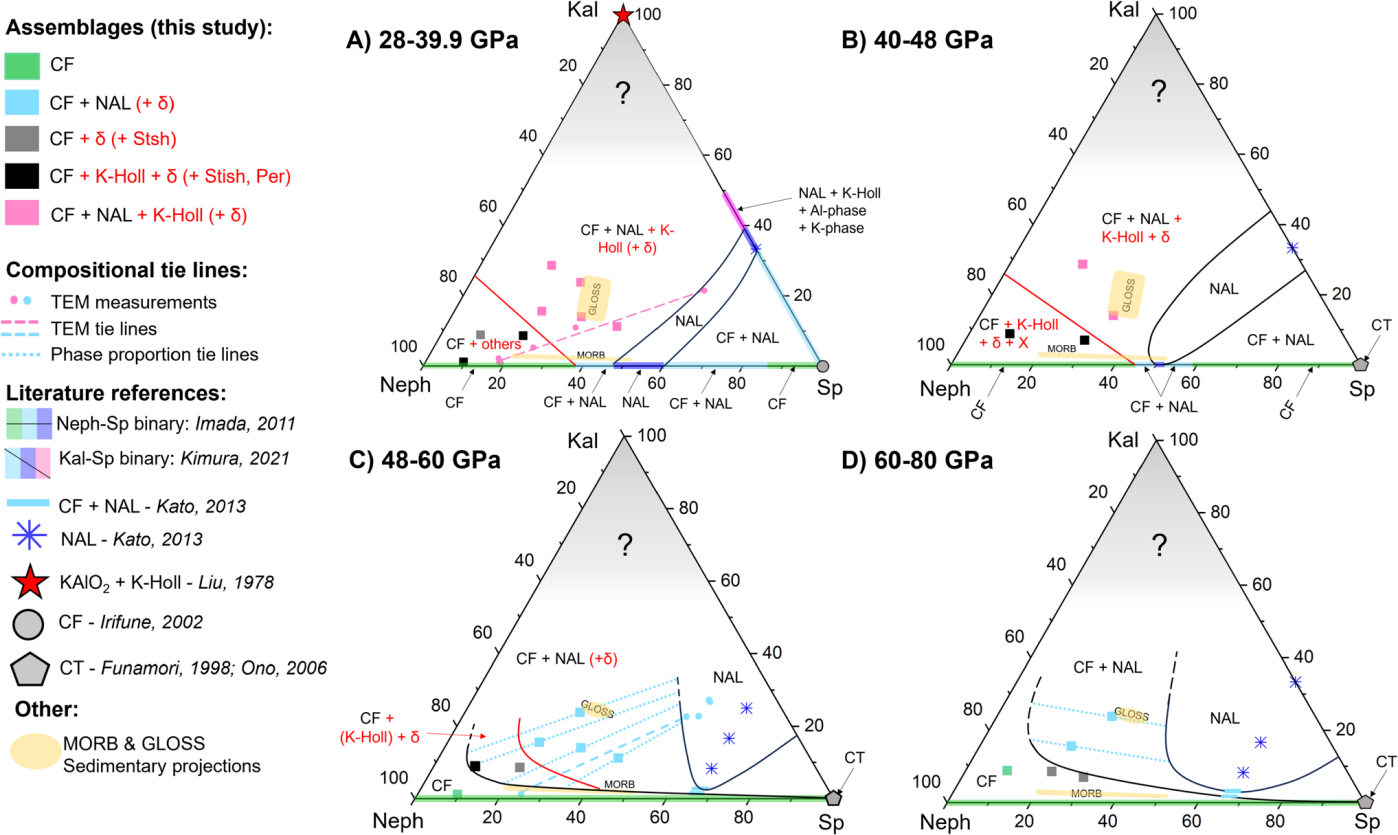
Phase relations on the nepheline-spinel-kalsilite compositional join as a function of pressure based on this study and data from the literature. Colours indicate the observed phase assemblage, while shapes indicate the reference. Dashed lines indicate TEM tie lines between the NAL and CF compositions. Dotted lines indicate phase proportion tie lines, based on Rietveld refinements, angles of which were kept similar to the TEM tie line in C and to the microprobe tie line in D. Binary coloured axes are based on the phase assemblages of Imada et al. (2011) and Kimura et al. (2021). Yellow areas are possible compositional ranges for aluminous phases in sedimentary and MORB compositions. The greyed out areas are uncertain and were not studied here. Lines and writing in red indicate reactions and phases that lie outside of the ternary plane. Datapoints on the diagrams are displayed in mole %. Al-phases = $\delta$-phase and/or hydrous phase D; X: Stishovite or Periclase. K-phase was not further defined in Kimura et al. (2021). Kal = Kalsilite (KAlSiO_4_); Neph = Nepheline (NaAlSiO_4_); Sp = Spinel (MgAl_2_O_4_. Other references: Liu (1978b), Irifune et al. (2002), Funamori et al. (1998), Ono et al. (2006) and Kato et al. (2013)

We constructed the phase relations on the nepheline-spinel-kalsilite compositional join based on the phases observed in our experiments with additional compositional information from TEM and microprobe measurements, as well as relative phase proportions, as obtained by Rietveld refinements of five experiments. In our phase diagrams (Fig. 6), the studies of Imada et al. (2011), Kato et al. (2013) and Kimura et al. (2021) were used to anchor positions along set compositional lines and those of Liu (1978b), Funamori et al. (1998) and Ono et al. (2006) for end-member phases. We used the relative phase proportions for CF + NAL, as determined by Rietveld refinement in experiments where they co-exist, to construct the single phase stability fields. The angle of the tie lines was chosen to match those obtained from the TEM measurements for Fig. 6C. For the highest pressure region (Fig. 6D) the angle of the tie lines was obtained from fitting the microprobe data of the aluminous phases present in the run products of experiment 7-2 . Both TEM tie lines traverse two non-ternary phase fields separated by a phase boundary representing the reaction CF + K-Holl + $\delta$-phase = CF + NAL ± K-Holl ± $\delta$-phase at pressures below 50 GPa. The projected ranges of combined aluminous phases in MORB (Gale et al. 2013) and global subducting sediment (GLOSS; Plank and Langmuir 1998) are indicated as yellow areas. More information on the projections is provided in the supplementary information, Tables S4–S11 and Figure S3.

## Discussion

### Phase relations

The phase relations for the nepheline-spinel-kalsilite compositional join were evaluated based on this study and pre-existing data and were compiled in a series of phase diagrams for four different pressure brackets (Fig. 6). Phase relations with addition of a potassium-rich kalsilite component differ significantly from those for the pure nepheline-spinel boundary. The relations vary systematically with pressure, but follow a similar pattern at all pressures. Both CF and NAL have broad compositional ranges and display a large two phase field in between. At low pressure the K-poor end of the NAL stability field intersects the nepheline-spinel binary join. This K-poor tip shrinks and moves away from the join with increasing pressure. Simultaneously, the solubility of the kalsilite-component in NAL increases with increasing pressure. This allows NAL to maintain a significant stability field in the nepheline-kalsilite-spinel ternary system up to at least 71 GPa (Fig. 6). At 48 GPa there is a complete solid solution between the nepheline and spinel end-member for CF (Imada et al. 2011). We observe a broadening of the CF stability field towards higher potassium incorporation around the nepheline apex, while remaining limited towards the spinel apex. This is explicable considering the more similar ionic radii and identical charge of Na^+^- and K^+^-ions, when compared to Mg^2+^.

For the nepheline end-member it is well established that CF is the stable phase up to at least 75 GPa (Tutti et al. 2000). The spinel end-member undergoes a phase transition to $\epsilon$-MgAl_2_O_4_ (Liu 1978a; Enomoto et al. 2009), which remains uncharacterised (Ishii et al. 2021). A further phase transition to a Ca-Titanite (CT) type structure occurs at around 45 GPa (Funamori et al. 1998; Ono et al. 2006), which is expected to be stable up to at least 117 GPa (Ono et al. 2006). Neither of these phases are commonly reported for compositions deviating from the spinel apex, though CT has been observed for a MORB composition at 143 GPa (Ono et al. 2005). Here we assume negligible incorporation of a nepheline-component into the CT-structure up to pressures of 80 GPa (Fig. 6).

#### Implications of the presence of K-Hollandite as a reaction product

K-Hollandite was found to be part of the stable phase assemblage for a range of pressures and compositions. The phase has an ideal composition of KAlSi_3_O_8_ (Ringwood et al. 1967). The K-Hollandite analysed by TEM in experiment 6-4 has the composition K_0.6_Al_1.2_Si_3.0_O_8_ (see supplementary information, Figure S1). We observe K-Hollandite with pure CF, as well as with CF and NAL. Its presence indicates a kalsilite-component in excess of the solubility in the respective aluminous phases. In the presence of small amounts of water, e.g. through adsorbtion to the fine grained glass starting material during loading, the excess kalsilite-component may undergo the reaction:

1 $$\begin{aligned} 3\cdot \textrm{KAlSiO}_4 + \textrm{H}_2\textrm{O} \rightarrow \textrm{KAlSi}_3\textrm{O}_8 + 2\cdot \delta \textrm{AlOOH}\,[+\textrm{K}_2\textrm{O}]. \end{aligned}$$

For an anhydrous experiment this becomes:

2 $$\begin{aligned} 3\cdot \textrm{KAlSiO}_4 \rightarrow \textrm{KAlSi}_3\textrm{O}_8 + \textrm{Al}_2\textrm{O}_3\,[+\textrm{K}_2\textrm{O}]. \end{aligned}$$

Thus, the co-occurrence of K-Hollandite with not only pure CF, but also CF + NAL indicates a maximum potassium solubility not only for CF, but also for NAL. The excess K_2_O component was not observed and may have been lost during sample preparation.

We observe four main domains of behavioural shift with pressure:

(1): For the lowest pressure region from 28 to 39.9 GPa, the observed phase assemblages are by far the most complex. However, we find K-Hollandite to be a stable phase in almost all experimental compositions. Experiment 9-4 is an exception to this. We expect K-Hollandite has simply not been sampled, especially since for the same composition it is observed for the next higher pressure. The presence of K-Hollandite indicates that the potassium content is beyond the solubility limit of CF, and where applicable, also NAL, even for the most K-poor composition. Based on this we conclude that up to at least 33.1 GPa the solubility of potassium in CF is $<0.011(1)\frac{\textrm{K}}{\textrm{K}+\textrm{Na}+\textrm{Mg}}$.
(2): At pressures between 40 and 48 GPa K-Hollandite with $\delta$-phase was also found to coexist with CF, as well as with CF and NAL for all probed compositions, indicating that for this pressure range neither CF nor NAL have a sufficient capacity for potassium to account for the additional kalsilite component.
(3): For pressures between 48 and 60 GPa the most kalsilite-poor experimental composition (1.1(1) mole%) returns a pure CF phase assemblage. However, for a slightly larger kalsilite component, K-Hollandite is observed again. This indicates a potassium solubility in CF between $\frac{\textrm{K}}{\textrm{K}+\textrm{Na}+\textrm{Mg}}=0.011(1)$ and $\frac{\textrm{K}}{\textrm{K}+\textrm{Na}+\textrm{Mg}}=0.088(3)$, based on the respective starting material compositions. We do not observe K-Hollandite in experiments where both CF and NAL are stable, suggesting an increased potassium solubility in NAL.
(4): At pressures below 60 GPa for bulk compositions around the nepheline apex with up to 10 mole% nepheline, K-Hollandite is stabilized over NAL, with $\delta$-phase accounting for the excess alumina. For the highest applied pressures of 60–80 GPa we do not observe K-Hollandite in any of our experiments. K-Hollandite is known to be stable up to at least 95 GPa (Tutti et al. 2001). Its disappearance indicates that, due to increased potassium solubility in both CF and NAL, it is no longer energetically favourable for K-Hollandite to be part of the stable phase assemblage. Based on the starting material composition, we find CF to be able to incorporate a minimum of $\frac{\textrm{K}}{\textrm{K}+\textrm{Na}+\textrm{Mg}}=0.088(3)$ potassium at a pressure of 67.8 GPa. This is significantly higher than previously reported by Miyajima et al. (2001), Hirose and Fei (2002) and Ishii et al. (2022), but in agreement with the increasing K-solubility in CF with pressure, as observed by Kato et al. (2013).

### The presence of hydrous phases

Many of the experimental run products were found to contain hydrous phases. In most instances this was $\delta$-phase, but also hydrous phase D in two of the experiments. We do not observe any starting material composition dependence on the presence or absence of hydrous phases (Table 2). In any experiment where K-Hollandite was stabilised an additional high Al-phase is required to satisfy mass balance. $\delta$-phase and corundum both satisfy that condition. In experiments where K-Hollandite is observed, but neither $\delta$-phase or corundum, we argue that $\delta$-phase has not been sampled in the synchrotron XRD measurements, but is present in the phase assemblage, in order to satisfy the chemical balance of the assemblage. The abundance of $\delta$-phase in selected experimental run products was determined using Rietveld refinements (Table 3). In these cases, $\delta$-phase was found to comprise approximately 10 wt.% of the assemblage (Table 3). This is equivalent to 1.5 wt.% H_2_O, which is a very large amount, considering that all starting materials were stored in a 125 ^∘^C vacuum oven pre-loading. We assume that water was adsorbed by the very fine-grained and hence high-surface-area starting material after removal from the oven for the loading process. This could explain why hydrous phases are not observed in all experiments: the longer the time between removal of the starting material from the oven and loading the more moisture may be adsorbed. Notably, in later experiments using the same nepheline and kalsilite glasses, DACs were placed in a DAC-drying oven at 120 ^∘^C for an hour before being closed while within the oven at temperature. However, $\delta$-phase was still observed in some of the experimental run products. The presence of $\delta$-phase free experiments with pure CF or CF with NAL phase assemblages at pressures upwards of 50 GPa (Table 2) may indicate an increased solubility of water in the aluminous phases at higher pressures. The solubility of water in CF and NAL requires further investigation.

### Crystallographic considerations

We demonstrated above that CF may hold significant potassium at lower mantle pressures. Now we discuss how this impacts the lattice parameters of CF relative to NAL.

**Fig. 7:**
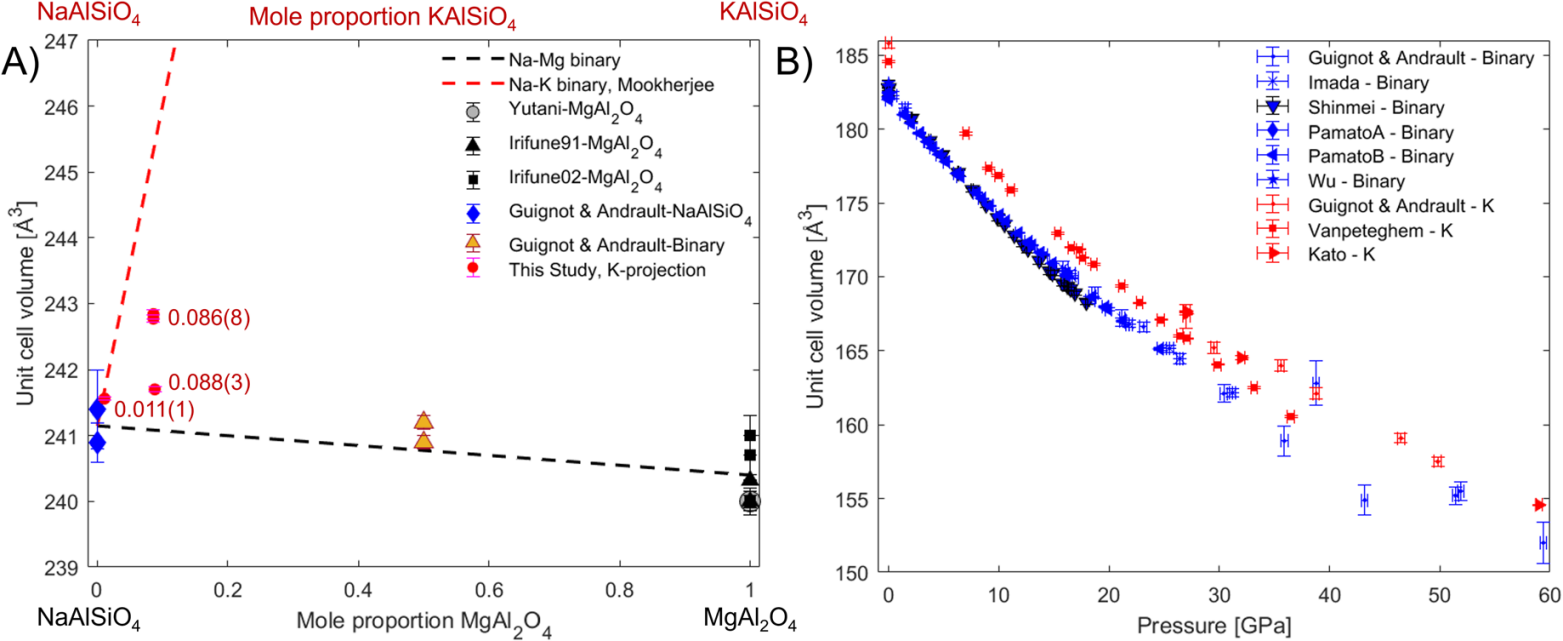
Impact of incorporation of K on unit cell volumes of NAL and CF. **A** Unit cell volumes of CF for different compositions at atmospheric pressure. The dashed black line is the mixing line between the average unit cell volume of CF for NaAlSiO_4_ and MgAl_2_O_4_ endmember compositions. The dashed red line is the mixing line between the experimental average unit cell volume of CF for the nepheline endmember with the theoretical kalsilite endmember, as determined by Mookherjee et al. (2012). The data from this study are projected onto the nepheline-kalsilite binary and the molar ratios are indicated for pure CF experiments in $\frac{K}{K+Na+Mg}$, assuming the starting material composition to be equivalent to the CF phase composition. The literature data is K-free, but occasionally contained additional components to nepheline and spinel. Where necessary, they were projected based solely on their nepheline and spinel contents. **B** Unit cell volumes of NAL with pressure. Blue symbols indicate data obtained for K-free compositions, red symbols indicate compositions containing K. CF Data: Irifune et al. (1991), Yutani et al. (1997), Irifune et al. (2002), Guignot and Andrault (2004); NAL Data: Vanpeteghem et al. (2003), Guignot and Andrault (2004), Shinmei et al. (2005), Imada et al. (2012), Kato et al. (2013), Pamato et al. (2014), Pamato (2014), Wu et al. (2016)

The compressibility of both CF and NAL is independent of the chemical composition of either phase for the compositions reported in the literature (supplementary information, Figure S4). For CF, there is also little compositional dependency of the unit cell volume on its location on the nepheline-spinel binary (Fig. 7A). The substitution of Mg^2+^ by Na^+^ lengthens the *a*-axis, whereas the substitution of Al^3+^ by Si^4+^ shortens the *c*-axis. The two effects almost balance each other out and return very similar unit cell volumes for the two end-members (Fig. 7A). It can be hypothesized that the substitution of K^+^ for Na^+^ lengthens the *a*-axis, while supplying no compensation in the *b*- or *c*-direction. Thus, the addition of a potassic component is expected to increase the *a/c* ratio, as well as the unit cell volume slightly over that reported for the NaAlSiO_4_ and MgAl_2_O_4_ end-members. This behaviour has been observed for the unit cell volumes of CF in the monophase experimental runs here (Fig. 7A, lattice parameters supplied in supplementary information, Table S12), where up to 8.8(3) mole% kalsilite-component were used in the starting materials. Our unit cell volumes for K-bearing CF fall above the nepheline-spinel binary, but below the nepheline-kalsilite mixing lines (Fig. 7A), which is reasonable, as we have more Mg than K for all of our experiments (Table 1). The trends for the nepheline and spinel end-members were found to persist upon compression. As no K-bearing equation of state for CF is reported, we can not evaluate the effect of K on CF with pressure.

For NAL, due to the lack of distinctive compositional end-members, chemical trends are not as easily analysed. However, we observe the unit cell volume of NAL to be significantly larger for K-bearing compositions, than for K-free compositions (Fig. 7B). Due to the stronger lengthening effect of K on the *a*-, than the *c*-axis, we observe significantly higher *a/c* axial ratios, for K-bearing compositions (see supplementary information, Figure S6). These trends were found to persist to high pressures.

### Implications

#### Aluminous phases can host alkali elements in lower mantle lithologies

*MORB.* In MORB compositions, previous studies predict that both CF and NAL will be stable at conditions of the topmost lower mantle (Ricolleau et al. 2010; Ishii et al. 2019) and pure CF from pressures upwards of about 50 GPa (Ricolleau et al. 2010; Imada et al. 2011). For higher pressures and compositions deviating from the nepheline-spinel binary, only Ricolleau et al. (2010) provide an insight to the respective aluminous phase stabilities. While we observe a large effect from the choice of assumptions for projected MORB aluminous phase compositions, we do observe similar trends to previous studies (Fig. 6). At pressures below 48 GPa the range of projections spans from the pure NAL via the CF+NAL binary to the pure CF phase field. The present aluminous phases appear to heavily depend upon the availability of Al, which is largely governed by bridgmanite and differences in bulk composition, which may explain discrepancies between observed aluminous phases at pressures below 48 GPa. At low pressures for an intermediate projection, MORB falls into the CF+NAL two phase field and shifts increasingly towards the pure CF stability field, indicating an increasing phase proportion of CF. At pressures greater than 48 GPa CF is the only stable aluminous phase in MORB compositions, for the whole range of projections and is therefore independent of bulk composition and Al-incorporation into bridgmanite. For pressures exceeding 48 GPa CF may contain the bulk of potassium contained in MORB compositions. At pressures below 48 GPa we expect NAL to contain the majority of the potassium and the coexisting CF to be quite K-poor, which is in agreement with earlier findings (Ono et al. 2001; Hirose and Fei 2002). Generally, the addition of the potassic component in amounts relevant to MORB compositions appears to have little effect on the stable aluminous phase assemblage, as the projections fall into roughly the same stability fields for potassium-bearing and pure nepheline-spinel binary compositions (Fig. 6).

*Sediments.* High pressure phase equilibria studies of sedimentary compositions at lower mantle conditions are rare. Ono (1998) investigated sedimentary compositions to pressures of 15 GPa, where neither CF nor NAL are expected to be stable. Rapp et al. (2008) report the stable phase assemblage for sedimentary compositions at 16–23 GPa to be K-Hollandite with stishovite, garnet, an Al-silicate and an Fe–Ti spinel. As garnet is still the stable Al-bearing phase, neither NAL nor CF were observed. Irifune et al. (1994) additionally observe calcium-perovskite and the onset of CF for their highest pressure point at 24 GPa. Ishii et al. (2012) find that for compositions of the continental crust, for pressures of 24–28 GPa, the stable phase assemblage is CF with Ca-perovskite, K-Hollandite and stishovite, observing CF as the main aluminous phase. This is similar to our findings for the lowest pressure region (Fig. 6). One previous study focused on a sedimentary composition at a range of lower mantle conditions (Armstrong et al. 2012). For the sedimentary composition they found CF with NAL, K-Hollandite and stishovite to be the stable phase assemblage above 30 GPa, with K-Hollandite disappearing between 40 and 50 GPa. The observations of these two studies are in general agreement with our findings. The presence of $\delta$-phase as an additional aluminous phase in place of stishovite is explained by the under-saturation of silicon in our experiments (Table 1), when compared to natural sedimentary compositions (Plank and Langmuir 1998). For the assumption of a K-Hollandite bearing sedimentary phase assemblage, which falls into the low K region of the projected GLOSS compositional range (Fig. 6A), the aluminous phases in GLOSS plot almost perfectly on the TEM compositional tie line between CF and NAL for experiment 6-4 (Fig. 6A). Based on this we expect approximately equivalent amounts of CF and NAL for sedimentary compositions. We can also deduce approximate chemical compositions for CF and NAL in average sedimentary compositions at $\approx$ 35 GPa, based on our STEM compositional measurements. In this case NAL would have a composition of (K_0.59_Na_0.52_)(Mg_1.65_Al_0.23_)(Al_4.9_Si_1.33_)O_12_ and the co-occurring CF would have a composition of (Na_0.70_Mg_0.20_K_0.03_)(Al_1.22_Si_0.85_)O_4_, though significant additional iron would be expected. We observe CF with NAL, K-Hollandite and an aluminous phase for pressures up to 48 GPa and just CF with NAL at pressures beyond that up to at least 80 GPa. With the broadening of the NAL stability field to higher pressures we may expect a higher phase proportion of NAL than of CF for pressures upwards of 60 GPa, also indicating that NAL is likely stable in sedimentary compositions throughout the entire depth of the lower mantle. For sedimentary compositions at pressures below 50 GPa we expect the bulk of the kalsilite component to be held in the K-Hollandite phase, with NAL containing up to 25 mole% kalsilite and negligible potassium in CF (Fig. 4).

*K-Hollandite* has previously been mentioned to be a suitable host for potassium in sedimentary compositions in the lower mantle (Guignot and Andrault 2004; Hirao et al. 2008), as it can accommodate potassium well in its large tunnel space surrounded by octahedral chains (Ishii et al. 2019). However, it has also previously been argued that the stabilization of K-Hollandite leads to the absence of CF and NAL, as it takes large amounts of the alumina component (Ishii et al. 2019). Here we show that CF and NAL can coexist with K-Hollandite in the nepheline-kalsilite-spinel compositional join (Table 2, Fig. 6) and are expected to be stable in sedimentary compositions up to pressures of 48 GPa. In more silica-enriched compositions K-Hollandite may be the stable aluminous phase (Tutti et al. 2001), but is not commonly observed for more Si-rich experiments on MORB compositions at lower mantle conditions (Ricolleau et al. 2010; Ishii et al. 2019).

**Fig. 8:**
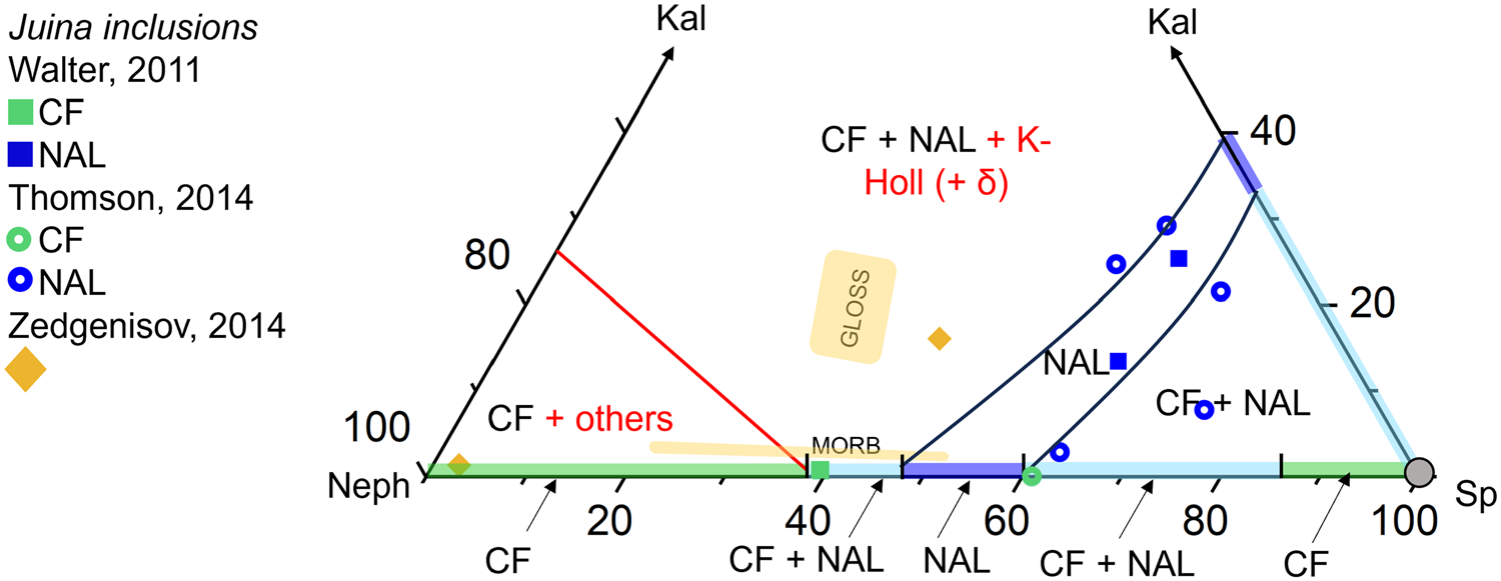
Aluminous phase compositions as measured from diamond inclusions. Juina diamond inclusions are reported in Walter et al. (2011), Thomson et al. (2014) and Zedgenizov et al. (2014). The inclusion compositions are projected based on their sodium and potassium contents for nepheline and kalsilite respectively. We project MgO, MnO and CaO contents of the inclusion onto the spinel apex. The phase relations for the nepheline-spinel-kalislite compositional join are displayed for conditions of the uppermost lower mantle (28–39.9 GPa). Red lines and text indicate reactions and phases that fall outside of the ternary. We also display MORB (Gale et al. 2013) and GLOSS (Plank and Langmuir 1998) compositional projection ranges (yellow areas). For details see the supplementary information, Tables S4–S11 and Figure S3

*Inclusions in diamonds.* When comparing our phase relation results with phase compositions obtained from inclusions in diamonds (Thomson et al. 2014; Zedgenizov et al. 2014), we find a reasonable agreement between the inclusion data and our constructed phase relations (Fig. 8). The aluminous phases in all studies were found to have transformed to composite inclusions of spinel, nepheline, kalsilite or clinopyroxene upon exhumation. In many cases not all of these breakdown products were identified and measured, contributing to uncertainty over the compositions of the reconstructed phases (Walter et al. 2011; Thomson et al. 2014; Zedgenizov et al. 2014). The bulk of the inclusions assigned as NAL in Thomson et al. (2014) and all NAL inclusions of Walter et al. (2011) fall into or close to the NAL stability field for the topmost lower mantle. The inclusion assigned as CF in Walter et al. (2011) falls onto the very tip of the CF phase stability field, whereas the inclusion assigned as CF in Thomson et al. (2014) falls onto the edge of the NAL field (Fig. 8). As only the presence of potassium in NAL was used as a distinction between CF and NAL that disagreement is only of minor concern, as NAL can also be stabilized in K-free compositions at conditions of the topmost lower mantle (Imada et al. 2011). Thus, the pure compositional distinction between the two phases hinders further interpretation of the agreement of diamond inclusion data with the phase relations, as phase identities remain ambiguous. The inclusions characterized by Zedgenizov et al. (2014) appear significantly more scattered. For the inclusion close to the nepheline apex only the nepheline component was identified, potentially leading to an inaccurate composition. The inclusion with both nepheline and spinel components measured falls within the range of GLOSS projections and into the central CF+NAL bi-phase field (Fig. 8).

## Conclusions

We demonstrate that the presence of small amounts of a kalsilite-component allows NAL to persist to higher pressures than for the pure nepheline-spinel binary join (Imada et al. 2011). In such potassium-bearing compositions, we find NAL to be a stable aluminous phase up to at least 71 GPa. For CF on the other hand, only minor amounts ($\frac{\textrm{K}}{{K}+{Na}+{Mg}}=0.011(1)$) of potassium can be incorporated into CF below 50 GPa. This solubility was found to increase with pressure and at 68 GPa we observe a minimum K solubility of at least $\frac{\textrm{K}}{K+Na+Mg}=0.088(3)$ in CF. We conclude that the expected stable phase assemblage of aluminous phases can hold the alkali content expected for MORB compositions in the lower mantle. For sedimentary compositions at lower mantle pressures, we expect both CF and NAL to be stable with K-Hollandite for pressures of 28–48 GPa and just CF with NAL for pressures of 48–77 GPa. In sedimentary compositions, NAL may be stable alongside CF throughout the entire lower mantle and it may even be the dominant aluminous phase upwards of 60 GPa.

### Supplementary Information

Below is the link to the electronic supplementary material.

Supplementary file 1 (pdf 5243 KB)

## Data Availability

The data underlying this article are available at the University of Bristol data archive. The repository contains integrated diffraction patterns for each successful experiment as well as temperature logs of the laser heating runs. Data are available at the University of Bristol data repository, data.bris, at https://doi.org/10.5523/bris.1pc3wtlhcim8q2ajm7bmoa9dfw.
