# Supplementary material for: The effect of potassium on aluminous phase stability in the lower mantle
Source: Contrib Mineral Petrol. 2024 Apr 27;179(5):52. doi: 10.1007/s00410-024-02129-w (PMC11055704; doi:10.1007/s00410-024-02129-w)
Supplement: Supplementary file 1 — (pdf 5243 KB) [file 410_2024_2129_MOESM1_ESM.pdf]

Supplementary information for: The effect of potassium on aluminous phase stability in the lower mantle

Elena-Marie Rogmann [a], Eleanor S. Jennings [a,b*], Jennifer Ross [a], Nobuyoshi Miyajima[b], Michael J. Walter[a,†], Simon C. Kohn[a], Oliver T. Lord [a]

[a] School of Earth Sciences, University of Bristol, Bristol, UK
[b] Bayerisches Geoinstitut, Universität Bayreuth, Bayreuth, Germany
[*] Now: School of Natural Sciences, Birkbeck, University of London, London, UK
[†] Now: Earth and Planets Laboratory, Carnegie Institution for Science, Washington DC, USA
Corresponding author e-mail: e.rogmann@bristol.ac.uk

Contents

In this supplementary information file we supply additional figures and data tables, and provide information on the ternary projections of the MORB and GLOSS compositions. Tables S1 and S3 provide the compositions determined from the FEG EPMA measurements on the biphase experiments 1-2, 1-3 and 7-2 (Table S1), the monophase experiments 8-2 and 9-6 (Table S2) and TEM measurements on the phases in experiments 2-3 and 6-4 (Table S3), as projected onto the nepheline-kalsilite-spinel ternary. We also supply additional STEM images of different phases with their respective selected area diffraction patterns (SAED) (Figure S1).

We display the relative phase abundances for eight experiments, as obtained by Rietveld refinement (Figure S2). These exhibit increasing CF abundance towards more nepheline-rich bulk compositions, while we observe a decrease towards bulk compositions richer in the kalsilite and spinel components. We find the abundance of the δ-phase, to be uniformly low at around 10 % and similar to K-Hollandite. There is significant scatter in the abundance of the NAL component, with a slight trend to higher NAL proportions with increasing kalsilite contents (Figure S2).

We provide information on the projections of MORB and GLOSS under different assumptions in the section 'Calculation of MORB and GLOSS projections', containing Tables S4 through S11, with an overview of the process provided in Figure S3.

To supplement the discussion we provide an array of crystallographic comparisons. We present the evolution of the normalized unit cell volumes of CF and NAL with pressure (Figure S4) to show that the compression mechanism of neither phase is composition dependent, for the range of compositions studied so far. We also supply ambient pressure unit cell dimensions of CF determined here (Table S12), in comparison to values reported in the literature (Figure S5). We find that the incorporation of potassium appears to have the strongest impact on the a-axis, whereas results for the c-axis fall onto the nepheline-spinel mixing line. Lastly, we compare the a/c ratio of CF and NAL (Figure S6) and their compositional dependence. The addition of potassium was found to significantly increase the a/c ratio of NAL. This increase was positively correlated with the K-content of the NAL phase. Due to lack of in situ unit cell parameter data of CF, containing K, we can only analyze the effect of other components. We find that the nepheline endmember has a significantly higher axial ratio than the spinel endmember, while intermediate compositions can be described by linear mixing. Based on the increase of the unit cell volume of CF in the presence of K (see main document) and the stronger effect of K on the a-axis compared to the c-axis (Figure S5), we can hypothesize an axial ratio for K-bearing CF even larger than for the nepheline endmember (Figure S6).

Table S1: FE EPMA measurements on the CF and NAL bearing experiments (Exp.) CAFHP 1-2 (50 GPa), 1-3 (65 GPa) and 7-2 (71 GPa). Oxides are given in weight %, ternary endmember projections are provided in mole %. Different measurement points (Pt.) indicate measurements at different locations on the sample. Neph = Nepheline; Kal = Kalsilite; Sp = Spinel.

Exp.	Point	SiO_2	Al_2O_3	MgO	Na_2O	K_2O	PtO	Total	Neph	Kal	Sp
1-2	1	28.82	43.18	5.58	11.46	5.58	1.19	95.81	59	62	62
	2	29.54	41.24	5.00	12.70	5.76	0.81	95.05	19	19	18
	3	29.74	41.76	5.14	12.52	5.60	0.34	95.10	22	19	20
1-3	2	26.79	44.84	6.83	12.16	4.89	0.53	96.04	59	16	25
	3	28.76	41.4	4.99	14.42	5.56	0.21	95.34	66	17	18
	5	30.01	42.51	4.75	14.23	5.28	0.38	97.17	67	16	17
	6	27.52	42.23	6.61	13.13	5.07	0.8	95.36	61	15	24
	7	29.97	41.96	5.02	12.17	5.2	0.87	95.18	63	18	20
	8	28.67	41.93	4.87	14.69	5.35	1.2	96.71	67	16	17
	9	31.16	40.52	3.91	15.03	5.35	0.86	96.83	70	16	14
	13	30.94	38.92	4.23	11.54	5.7	3.7	95.04	62	20	18
	14	31.81	40.83	4.81	13.94	5.1	0.71	97.2	66	16	18
	15	32.48	42.4	4.25	13.68	6.02	0.58	99.41	65	19	16
	24	32.41	42.53	5	11.08	6.92	0.86	98.8	57	23	20
	25	31.39	41.82	6.47	8.18	5.33	3.09	96.28	49	21	30
	26	34.43	41.78	5.18	10.99	7.38	0.05	99.8	55	24	20
	27	31.03	43.67	6.1	9.62	6.37	1.45	98.24	52	23	25
	29	32.96	45.14	5.66	9.29	5.86	0.74	99.65	53	22	25
	30	32.09	41.23	5.46	13.8	6.46	1	100.03	62	19	19
	31	31.88	40.02	5.42	14.87	6.04	1.28	99.51	65	17	18
	32	32.96	40.98	4.59	15.11	6.86	1.02	101.52	65	19	15
7-2	1	27.79	42.95	8.33	8.98	7.25	3.15	98.45	45	24	32
	2	25.57	48.45	9.61	8.73	7.37	1.03	100.53	42	23	35
	3	28.35	43.34	7.35	10.42	7.52	1.82	98.79	50	24	27
	4	27.41	45.79	8.14	10.62	7.39	1.76	100.11	49	22	29
	5	29.54	44.41	8.38	8.85	7.23	0.17	98.58	44	24	32
	6	32.04	39.84	5.08	11.06	9.17	2.24	99.42	53	29	19
	7	30.52	41.28	7.02	9.16	7.70	0.35	96.03	47	26	28
	8	22.21	49.84	12.00	7.87	6.14	0.09	98.15	37	19	44
	9	28.36	39.62	5.29	10.65	7.27	6.11	97.31	55	25	21
	10	26.87	44.65	8.27	8.16	7.01	2.77	97.73	43	24	33
	11	26.39	47.85	9.14	9.04	7.97	0.14	100.54	42	25	33
	13	34.11	42.59	5.27	7.99	7.71	1.62	99.30	47	30	24
	14	27.09	46.74	7.70	7.40	6.74	0.87	96.54	42	25	33
	15	27.40	42.08	5.22	9.19	7.21	4.15	95.25	51	26	22
	16	25.39	47.40	8.02	9.13	7.88	0.28	98.11	45	25	30
	17	26.08	45.52	7.62	9.73	7.48	0.51	96.94	47	24	29
	18	27.72	41.61	7.21	7.31	6.23	6.68	96.76	43	24	33
	19	23.97	45.34	9.21	6.82	6.73	5.96	98.03	37	24	39
	20	31.05	41.55	4.59	11.58	8.92	1.74	99.44	55	28	17
	21	23.77	50.34	10.71	8.46	7.18	1.33	101.79	39	22	38
	22	30.08	42.55	5.83	10.39	8.29	0.81	97.94	51	27	22
	24	30.26	42.81	5.97	10.27	8.62	0.15	98.08	50	28	22
	25	31.77	41.80	5.27	10.66	9.34	1.34	100.18	51	29	19
	27	26.73	47.01	9.22	8.33	7.58	1.13	100.00	41	24	35
	28	28.36	43.28	7.15	9.33	8.15	1.73	98.01	46	27	27
	29	25.68	46.52	8.13	7.82	7.44	0.96	96.55	41	26	33
	30	33.70	40.52	4.74	11.13	8.93	0.81	99.84	54	28	18
	31	22.63	48.83	11.06	6.41	6.91	1.61	97.47	33	23	44
	32	26.80	44.69	8.73	8.89	7.94	1.57	98.62	43	25	32

Table S2: FE EPMA measurements on the CF monophase experiments (Exp.) CAFHP 8-2 (54 GPa) and 9-6 (68 GPa). Oxides are given in weight %, ternary endmember projections are provided in mole %. Different measurement points (Pt.) indicate measurements at different locations on the sample. Neph = Nepheline; Kal = Kalsilite; Sp = Spinel.

Exp.	Point	SiO_2	Al_2O_3	MgO	Na_2O	K_2O	PtO	Total	Neph	Kal	Sp
8-2	2	37.26	38.54	3.14	18.31	0.24		97.49	87.7	0.8	11.6
	3	36.91	37.02	2.95	20.04	0.07		96.99	89.6	0.2	10.1
	4	37.93	38.32	2.74	18.18	0.37		97.55	88.6	1.2	10.3
9-6	2	36.34	38.15	2.95	15.67	3.03	1.57	97.7	79	10	11
	5	37.6	37.94	2.35	14.25	2.73	1.25	96.11	80	10	10
	6	39.45	38.73	2.96	13.79	1.74	1.32	98	80	7	13
	7	38.92	39.47	2.74	14.59	3.32	0.18	99.22	77	12	11
	8	36.26	40.63	3.61	13.85	1.99	1.48	97.82	77	7	15

Table S3: TEM compositional data for the experiments 2-3 and 6-4 projected onto the ternary defined by nepheline (neph), kalsilite (kal) and spinel (sp) endmembers. Exp.: Experiment; P: Pressure; T: Temperature.

Exp.	P [GPa]	T [K]	Point	Neph [mole%]	Kal [mole%]	Sp [mole%]
2-3	56.1	2061	4	74	1.3	24.7
2-3	56.1	2061	8	23.9	22.7	53.5
2-3	56.1	2061	1	16.2	27.3	56.5
2-3	56.1	2061	1	16.2	26.8	57
2-3	56.1	2061	11	20.9	22.8	56.3
6-4	34.7	1992	Map2-3	69.7	5.3	24.9
6-4	34.7	1992	Map2-5	56.3	11	32.7
6-4	34.7	1992	Map1-1	80	2.2	17.8
6-4	34.7	1992	Map1-2	80.1	0.9	19
6-4	34.7	1992	Map1-3	80.4	1.1	18.5
6-4	34.7	1992	Map1-6	18.9	21.5	59.7

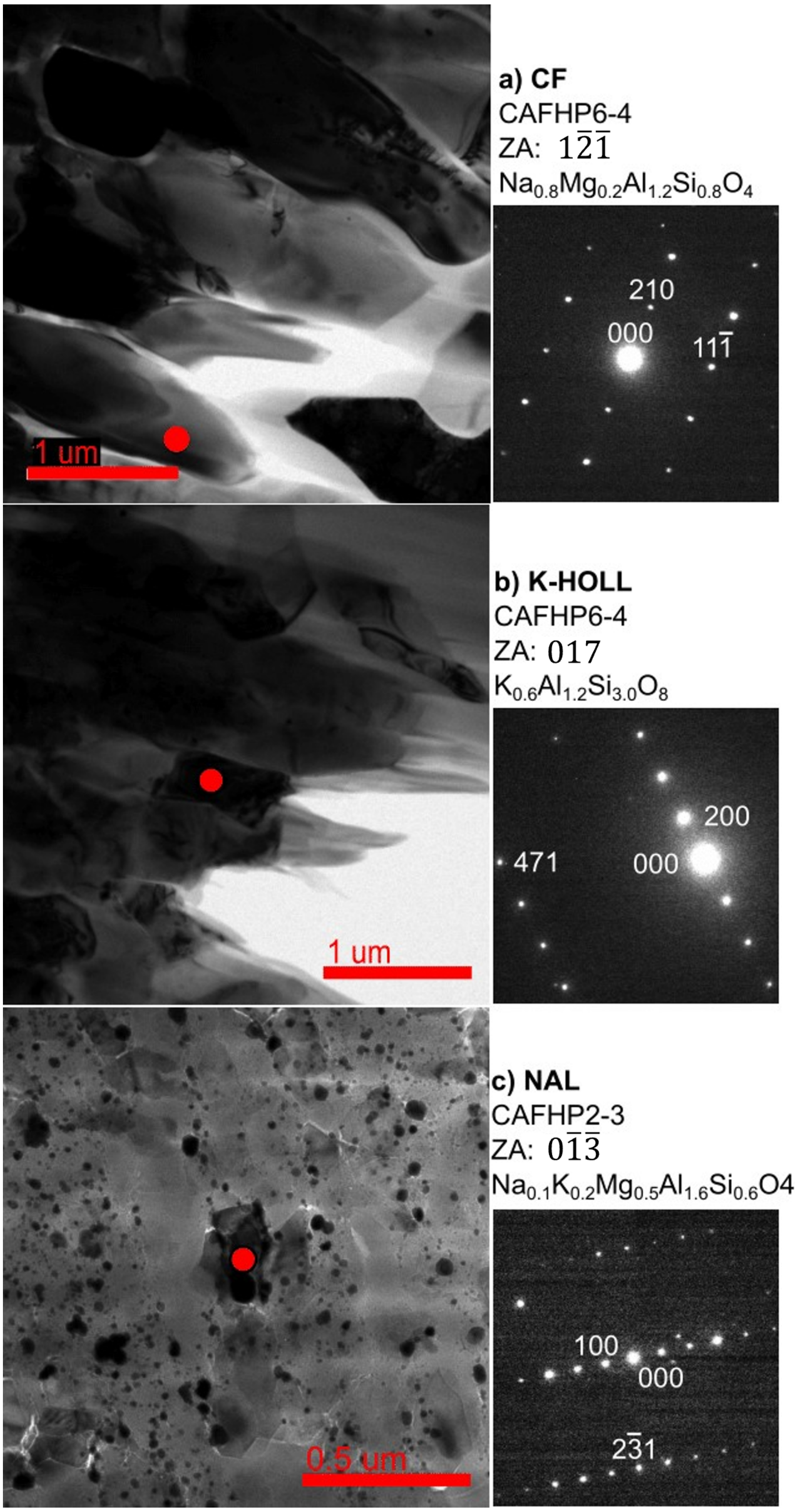

Figure S1: STEM images of experiment CAFHP 6-4 (A,B) and CAFHP 2-3 (C) focused on different phases. A) CF-phase, bright field (BF) STEM image and selected area diffraction (SAED) patterns; B) K-Hollandite, High angle annual dark field (HAADF) STEM image and SAED patterns; C) NAL, BF STEM images and SAED patterns. The red dots indicated the areas chosen for SAED. ZA: zone-axis.

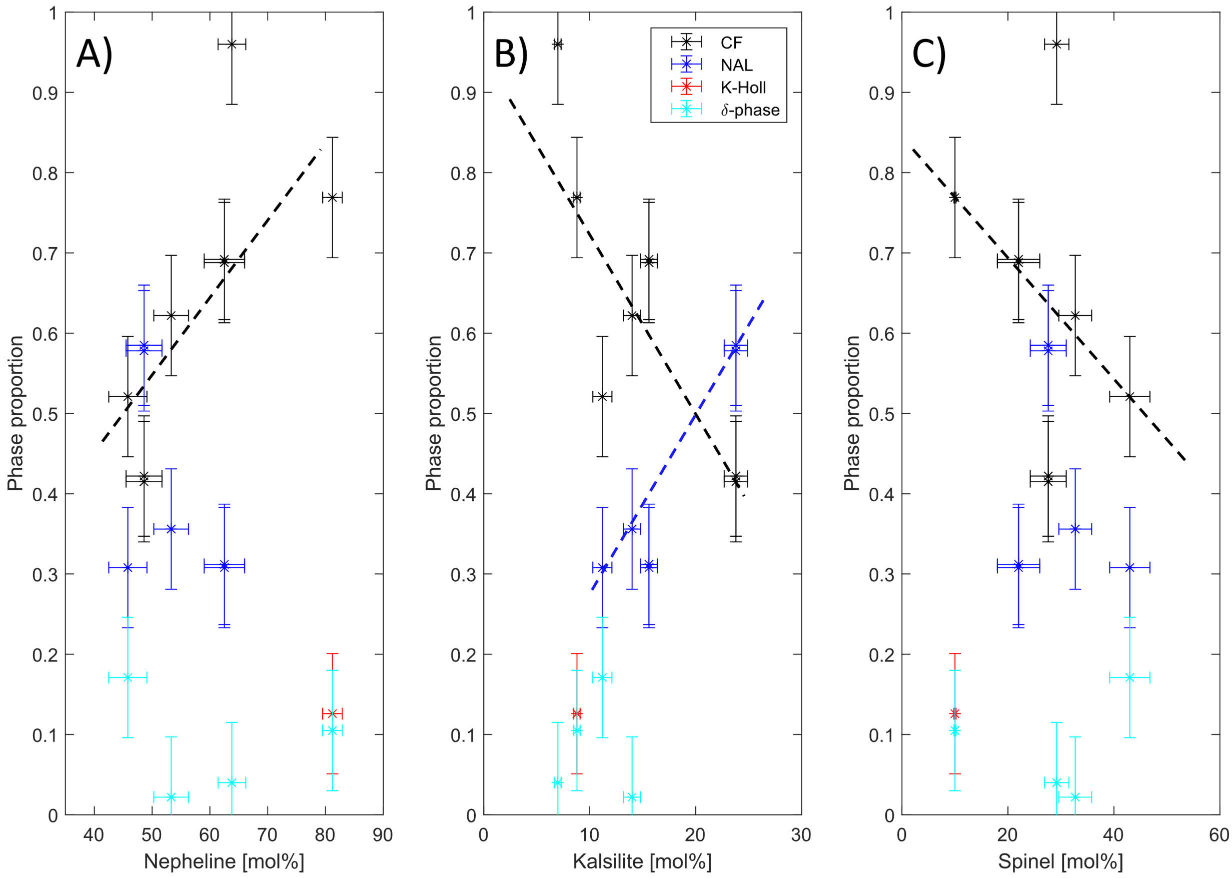

Figure S2: Relative phase proportions of CF, NAL, K-Hollandite and δ-phase, as a function of composition. Uncertainties are estimated at 15 % to account for the fact that the samples are not ideal powders. Phase proportions as a function of (A) nepheline content, (B) kalsilite content and (C) spinel content of the starting material.

Calculations of MORB and GLOSS projections

MORB (Gale et al. 2013) and GLOSS (Plank and Langmuir 1998) compositions were projected onto the nepheline-kalsilite-spinel compositional join based on their SiO_2-, Al_2O_3-, MgO-, CaO-, MgO-, FeO-, K_2O- and Na_2O-contents. A summary of the processes conducted to obtain the different aluminous phase projections is provided in Figure S3. To cover a reasonable array of possible aluminous phase compositions for the two lithologies, we first projected mean GLOSS and MORB. Next, we varied, in turn, the K, Na, Mg and Al content of each lithology to the maximum and minimum value allowed within the uncertainty on the mean reported for that element. The composition was then re-normalised and projected onto the ternary. This was repeated twice for each lithology, using different assumptions about the distribution of components amongst the stable phases, as discussed below. This procedure results in 16 points for each lithology, which are used to generate the fields drawn on the ternaries in Figure 6 of the main text and encompass the likely range of aluminous phase compositions in the lower mantle. As the compositional variation of GLOSS is significantly larger than that of MORB, we used 1 σ uncertainty for GLOSS and 2 σ uncertainty for MORB for our compositional assumptions (Table S4). The following example calculation will apply the mean compositions of MORB and GLOSS (Tables S4-S10), but ternary projections of all assumptions are listed in Table S11.

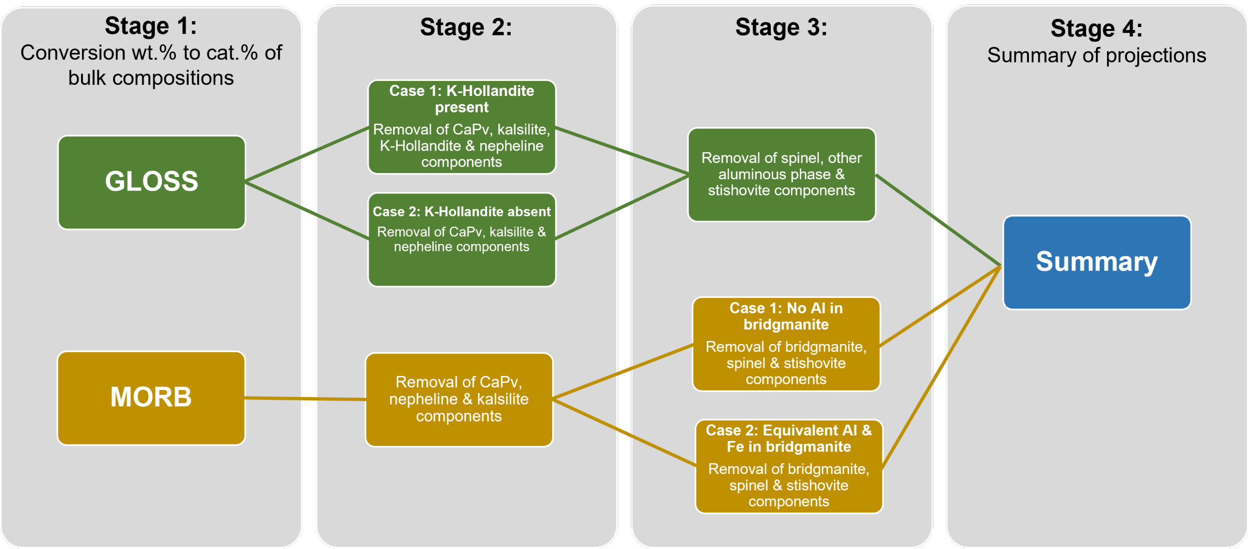

Figure S3: Flow chart of steps in the calculations to obtain the different projection endmembers for aluminous phase compositions as obtained from GLOSS and MORB bulk compositions in the nepheline-kalsilite-spinel system. CaPv = Calcium-perovskite.

Stage 1. Conversion of weight percent of major oxides to cation percent.

Table S4: Conversion of MORB (Gale et al. 2013) and GLOSS (Plank and Langmuir 1998) compositions from weight % to cation % with 2 σ uncertainty for MORB and 1 σ uncertainty for GLOSS, as the compositional variation is stronger.

	MORB wt.%	2 σ		Cation %		GLOSS wt.%	1 σ		Cation %
SiO_2	50.47	0.08	Si	47.78	SiO_2	58.57	2.49	Si	62.08
Al_2O_3	14.7	0.12	Al	16.40	Al_2O_3	11.91	0.94	Al	14.88
MgO	7.58	0.12	Mg	10.70	MgO	2.48	0.13	Mg	3.92
FeO	10.43	0.21	Fe	8.26	FeO	5.21	0.42	Fe	4.62
CaO	11.39	0.09	Ca	11.55	CaO	5.95	1.75	Ca	6.76
Na_2O	2.79	0.03	Na	5.12	Na_2O	2.43	0.2	Na	4.99
K_2O	0.16	0.014	K	0.19	K_2O	2.04	0.16	K	2.76
Total	97.52			100	Total	88.59			100

MORB Projections

For MORB we assume that all Na_2O and the required amounts of Al_2O_3 and SiO_2 go into the nepheline component. We make the same assumption regarding K_2O and kalsilite. For the spinel component there are two possible assumptions. One assumption is that the bulk of the alumina goes into the aluminous phases and is the limiting factor for the spinel component. Another assumption is that significant alumina goes into bridgmanite. When considering that both Fe and Al are expected to comprise about 0.15 cation% of the bridgmanite phase (Ishii et al. 2022), we can assume that about the same amount of Al as Fe goes into bridgmanite, leaving the remaining alumina component as the limiting factor for the spinel component. However, Ricolleau et al. (2010) argue for an even higher alumina content in bridgmanite. Excess MgO, FeO and SiO_2 as well as CaO crystallise bridgmanite, stishovite and Ca-silicate perovskite, though iron may also be present in the spinel component of CF and NAL. See below for the procedure of the respective calculations:

MORB Stage 2. Removal of Ca-perovskite, nepheline and kalsilite components. For Ca-perovskite the Ca component and an equal amount of Si are removed for an ideal composition. For nepheline and kalsilite the limiting components are sodium and potassium respectively. Silicon and alumina are removed in equal parts for both (Table S5).

Table S5: Removal of the Ca-perovskite, nepheline and kalsilite components from the MORB composition.

Si	Cation %	Ca-Perovskite	remain	Nepheline	remain	Kalsilite	remain
Si	47.78	11.55	36.23	5.12	31.11	0.19	30.91
Al	16.40	-	16.40	5.12	11.28	0.19	11.09
Ca	11.55	11.55	0.00	-	0.00	-	0.00
Fe	8.26	-	8.26	-	8.26	-	8.26
Mg	10.70	-	10.70	-	10.70	-	10.70
Na	5.12	-	5.12	5.12	0.00	-	0.00
K	0.19	-	0.19	-	0.19	0.19	0.00
Total	100.00	23.10	76.90	15.36	61.53	0.58	60.95

MORB Stage 3, Case 1: No Al in bridgmanite. Additional removal of bridgmanite, spinel and stishovite component. In this case we assume that all alumina goes into the aluminous phases CF and NAL, or K-Hollandite. First all alumina and magnesium in a 2:1 molar ratio are removed for the spinel component. The remaining magnesium and iron then dictates how much silicon is removed from the mix alongside them to form a bridgmanite phase. The remaining silicon is assumed to go into a silica phase (Table S6).

Table S6: Case 1 (MORB): No Al in bridgmanite, removal of spinel, bridgmanite and stishovite component for a MORB composition.

	Cation %	Spinel	remain	Bridgmanite	remain	Stishovite	final
Si	30.91	-	30.91	13.41	17.50	17.50	0.00
Al	11.09	11.09	0.00	-	0.00	-	0.00
Ca	0.00	-	0.00	-	0.00	-	0.00
Fe	8.26	-	8.26	8.26	0.00	-	0.00
Mg	10.70	5.54	5.15	5.15	0.00	-	0.00
Na	0.00	-	0.00	-	0.00	-	0.00
K	0.00	-	0.00	-	0.00	-	0.00
Total	60.95	16.63	44.32	26.82	17.50	17.50	0.00

MORB Stage 3, Case 2: Equivalent Fe and Al in bridgmanite. Additional removal of bridgmanite, spinel and stishovite component. In this case we assume that equal amounts of Fe and Al go into the bridgmanite phase (Ishii et al. 2022), both of which are removed initially. The remaining Al, together with Mg in a 2:1 ratio are removed to form the spinel component. The remaining Mg is assumed to go into bridgmanite and the amount of Mg and Fe in excess of Al is deducted in Si for the bridgmanite component. Lastly, all remaining Si is assumed to for stishovite (Table S7).

Table S7: Case 2 (MORB): Equivalent Al and Fe in bridgmanite, removal of spinel, bridgmanite and stishovite component for a MORB composition. Cat.: Cation; Brdg: Bridgmanite; Stsh: Stishovite; Tot.: Total.

	Cat. %	Brdg Pt.1	remain	Spinel	remain	Brdg Pt.2	remain	Stsh	final
Si	30.91	-	30.91	-	30.91	7.87	23.05	23.05	0.00
Al	11.09	8.26	2.83	2.83	0.00	-	0.00	-	0.00
Ca	0.00	-	0.00	-	0.00	-	0.00	-	0.00
Fe	8.26	8.26	0.00	-	0.00	-	0.00	-	0.00
Mg	10.70	-	10.70	1.41	9.28	9.28	0.00		0.00
Na	0.00	-	0.00	-	0.00	-	0.00	-	0.00
K	0.00	-	0.00	-	0.00	-	0.00	-	0.00
Tot.	60.95	16.51	44.44	4.24	40.20	17.15	23.05	23.05	0.00

GLOSS Projections

For GLOSS projections we base the stable phase assemblage at lower mantle conditions on the studies conducted by Ishii et al. (2012) and Armstrong et al. (2012). Ishii et al. (2012) find that up to pressures of at least 26 GPa aluminous phases coexist with K-Hollandite, Ca-perovskite and stishovite. Armstrong et al. (2012) observe a similar assemblage without Ca-perovskite and K-Hollandite, which disappears at pressures between 40 and 50 GPa. Based on these findings we use a phase assemblage of aluminous phases, Ca-perovskite, stishovite and K-Hollandite for our projections. We assume the absence of K-Hollandite for our ternary diagrams upwards of 48 GPa and for lower pressure range supply a range of compositions assuming the absence and presence of K-Hollandite. When K-Hollandite is present, we assume a 1:1 distribution of potassium between K-Hollandite and the aluminous phases, as K-Hollandite and CF are expected in approximately equivalent abundances at topmost lower mantle conditions for sedimentary compositions (Ishii et al. 2012).

GLOSS Stage 2, Case 1: K-Hollandite present. Removal of Ca-perovskite, kalsilite, K-Hollandite and nepheline components. In this case we assume that potassium is in equal parts incorporated into the kalsilite component and K-Hollandite. As a first step, we remove the Ca-perovskite component based on the availability of Ca and remove Si in equal parts. Subsequently we assign half of the present potassium to the kalsilite phase and remove equivalent amounts of Al and Si to form the kalsilite component. The remaining potassium then goes into the K-Hollandite phase. We assume a composition of the K-Hollandite phase based on Ishii et al. (2012), for which K-Hollandite has the approximate composition $K_{0.8}Na_{0.1}Al_{1.2}Si_{2.9}O_8$ and remove the relative amounts of sodium, alumina and silicon from the mix. The remaining sodium and equivalent amounts of Al and Si are then removed to form the nepheline component (Table S8).

Table S8: Case 1 for the GLOSS composition. Removal of the Ca-perovskite (CapV), Kalsilite (Kal), K-Hollandite (K-Holl) and Nepheline (Neph) components. K-Hollandite and kalsilite were based on equivalent amounts of potassium for both phases. Cat.: Cation; Tot.: Totals.

	Cat.%	CaPv	remain	Kal	remain	K-Holl	remain	Neph	remain
Si	62.08	6.76	55.32	1.38	53.94	5.00	48.94	4.82	44.12
Al	14.88	-	14.88	1.38	13.50	2.07	11.43	4.82	6.61
Ca	6.76	6.76	0.00	-	0.00	-	0.00	-	0.00
Fe	4.62	-	4.62	-	4.62	-	4.62	-	4.62
Mg	3.92	-	3.92	-	3.92	-	3.92	-	3.92
Na	4.99	-	4.99	-	4.99	0.17	4.82	4.82	0.00
K	2.76	-	2.76	1.38	1.38	1.38	0.00	-	0.00
Tot.	100	13.51	86.49	4.14	82.35	8.62	73.73	14.46	59.27

GLOSS Stage 2, Case 2: K-Hollandite absent. Removal of Ca-perovskite, kalsilite and nepheline components. In this case we assume that all present potassium is soluble in the aluminous phases CF and NAL. As a first step, we remove the Ca-perovskite component based on the availability of Ca and remove Si in equal parts. Then, we remove the potassium and Si and Al in equal parts to create the kalsilite component. The same is applied for sodium and the nepheline component (Table S9).

Table S9: Case 2 for the GLOSS composition. Removal of the Ca-perovskite (CapV), Kalsilite and Nepheline components. We assume the absence of K-Hollandite

	Cation %	CaPv	remain	Kalsilite	remain	Nepheline	remain
Si	62.08	6.76	55.32	2.76	52.56	4.99	47.57
Al	14.88	-	14.88	2.76	12.12	4.99	7.13
Ca	6.76	6.76	0.00	-	0.00	-	0.00
Fe	4.62		4.62	-	4.62	-	4.62
Mg	3.92	-	3.92	-	3.92	-	3.92
Na	4.99	-	4.99	-	4.99	4.99	0.00
K	2.76	-	2.76	2.76	0.00	-	0.00
Total	100	13.51	86.49	8.27	78.21	14.98	63.23

GLOSS Stage 3: Removal of spinel, other aluminous phase and stishovite components. From the remainder we remove the spinel component, using the alumina as the limiting factor. The residual Mg, together with Fe, are part of an aluminous phase component outside of this ternary projection, yet of significant abundance (Ishii et al. 2012). They are removed with twice the amount of Si, assuming a (Mg,Fe)Si_2O_4 stoichiometry of the component. The remaining Si is assumed to form stishovite (Table S10).

Table S10: Removal of spinel, additional CF and stishovite components from GLOSS projections for the both cases. Case 1 assumes significant K-Hollandite is part of the stable phase assemblage, Case 2 assumes its absence. The limiting factor for the abundance of spinel in both cases is the availability of Al. Stsh: Stishovite; Tot.: Totals.

**Case 1:**	**K-Holl**						
	Cation %	Spinel	remain	Other CF	remain	Stsh	final
Si	44.12	-	44.12	10.46	33.66	33.66	0.00
Al	6.61	6.61	0.00	-	0.00	-	0.00
Mg	3.92	3.30	0.61	0.61	0.00	-	0.00
Fe	4.62	-	4.62	4.62	0.00	-	0.00
Ca	0.00	-	0.00	-	0.00	-	0.00
Na	0.00	-	0.00	-	0.00	-	0.00
K	0.00	-	0.00	-	0.00	-	0.00
Tot.	59.27	9.91	49.36	15.70	33.66	33.66	0.00
**Case 2:**	**no K-Holl**						
Si	Cation %	Spinel	remain	Other CF	remain	Stsh	final
Al	47.57	-	47.57	9.95	37.62	37.62	0.00
Mg	7.13	7.13	0.00	-	0.00	-	0.00
Fe	3.92	3.56	0.36	0.36	0.00	-	0.00
Ca	4.62	-	4.62	4.62	0.00	-	0.00
Na	0.00	-	0.00	-	0.00	-	0.00
K	0.00	-	0.00	-	0.00	-	0.00
Tot.	0.00	-	0.00	-	0.00	-	0.00
	63.23	10.69	52.54	14.92	37.62	37.62	0.00

Stage 4: Summary of Projections

Stage 4. Final normalized nepheline, kalsilite and spinel components: The respective obtained amounts for nepheline, kalsilite and spinel for both compositions and both respective conditions are summarised in Table S11.

Table S11: Normalised nepheline, kalsilite and spinel contents for MORB and GLOSS. For MORB we apply the the assumptions of Al-free and Al-bearing bridgmanite and calculate the resulting bulk aluminous phase composition. For GLOSS compositions we apply the assumptions of equivalent amounts of kalsilite and K-Hollandite or the case of absent K-Hollandite. We provide mean values, as well as values varying the amount of Na, K, Mg and Al to the maximum and minimum values of the uncertainty of the mean compositions of MORB and GLOSS. For each of these cases the amount of one element was changed, then all components were re-normalised subsequently. This provides a compositional array of aluminous phase compositions in MORB and GLOSS compositions at lower mantle conditions.

	Al in Brdg		[mole %]	Al-free Brdg		[mole %]
MORB	Neph	Kal	Sp	Neph	Kal	Sp
**Mean**	**76.10**	**2.87**	**21.02**	**47.16**	**1.78**	**51.06**
Min. Na	75.59	2.88	21.52	46.78	1.78	51.44
Max. Na	76.61	2.86	20.53	47.55	1.78	50.67
Min. K	76.20	2.62	21.18	47.20	1.63	51.17
Max. K	76.01	3.12	20.87	47.13	1.93	50.94
Min. Mg	76.10	2.87	21.02	47.16	1.78	51.06
Max. Mg	76.10	2.87	21.02	47.16	1.78	51.06
Min. Al	76.87	2.90	20.23	47.46	1.79	50.75
Max. Al	75.35	2.84	21.80	46.88	1.77	51.36
	K-Hollandite present		[mole %]	No K-Hollandite		[mole %]
GLOSS	Neph	Kal	Sp	Neph	Kal	Sp
**Mean**	**50.72**	**14.51**	**34.77**	**44.13**	**24.38**	**31.49**
Min. Na	47.43	14.83	37.74	41.25	24.83	33.92
Max. Na	53.88	14.20	31.91	46.91	23.94	29.14
Min. K	50.69	13.32	35.99	44.56	22.68	32.76
Max. K	50.76	15.70	33.53	43.72	26.04	30.24
Min. Mg	50.72	14.51	34.77	44.13	24.38	31.49
Max. Mg	50.72	14.51	34.77	44.13	24.38	31.49
Min. Al	54.06	15.47	30.47	46.55	25.71	27.74
Max. Al	47.77	13.67	38.56	41.96	23.17	34.87

Crystallographic data

Table S12: Lattice parameters of the CF phase as determined by Rietveld refinement for experiments (Exp.) that contained only CF or contained only minor δ-phase. XRD measurements were conducted on recovered gaskets and are, hence, atmospheric pressure volumes. The χ^2 values are an expression of the quality of the fit. Errors are estimated uncertainties on the Rietveld refinements.

Exp.	a [Å]	error	b[Å]	error	c[Å]	error	V[Å^3]	error	χ^2	Assemblage	
4-2	10.1578	0.0016	8.690	0.001	2.7511	0.0006	242.84	0.07	0.217	CF	δ-phase
4-3	10.157	0.001	8.670	0.001	2.7568	0.0005	242.77	0.06	0.072	CF	δ-phase
8-2	10.1543	0.0004	8.6564	0.0003	2.7481	0.0001	241.56	0.02	0.080	CF	
9-6	10.1470	0.0009	8.6599	0.0007	2.7506	0.0003	241.70	0.04	0.116	CF	

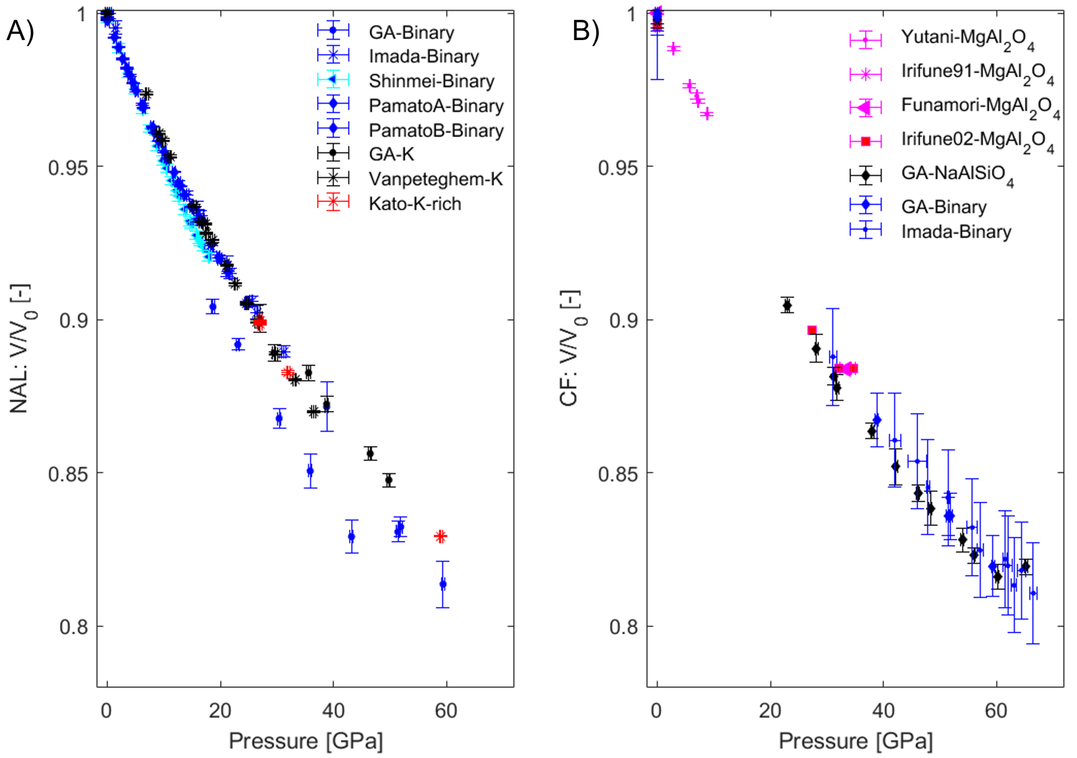

Figure S4: Normalized unit cell volume compression trends for A) NAL and B) CF. 'Binary' indicates that the phases' composition falls somewhere on the nepheline-spinel compositional binary; 'K' indicates that the phase contains small amounts of potassium, with 'K-rich' containing 10 mole% or more of kalsilite. References: Irifune et al. (1991); Yutani et al. (1997); Funamori et al. (1998); Irifune et al. (2002); Vanpeteghem et al. (2003); GA: Guignot and Andrault (2004); Shinmei et al. (2005); Imada et al. (2012); Kato et al. (2013); Pamato et al. (2014); Pamato (2014).

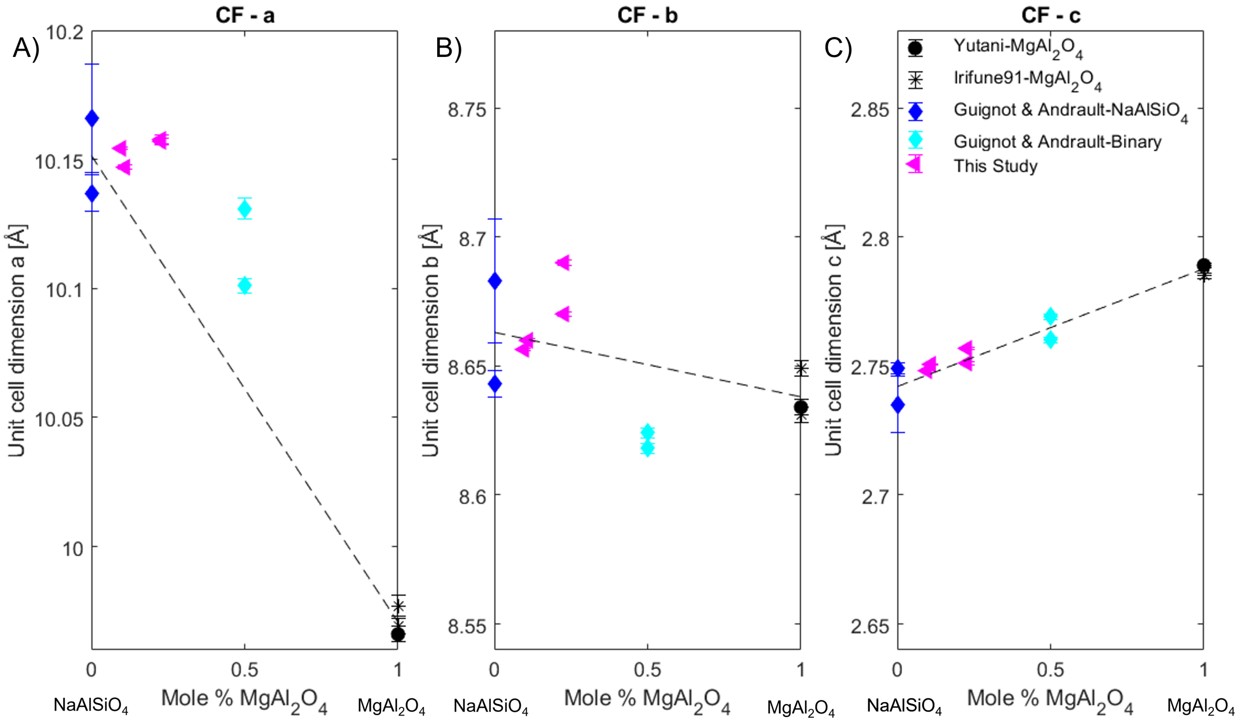

Figure S5: Ambient pressure unit cell dimensions of CF. A) a-axis; B) b-axis; C) c-axis; Binary indicates a phase composition that falls onto the nepheline spinel binary. The dashed lines are linear interpolations between the endmembers. References: Irifune et al. (1991); Yutani et al. (1997); Guignot and Andrault (2004).

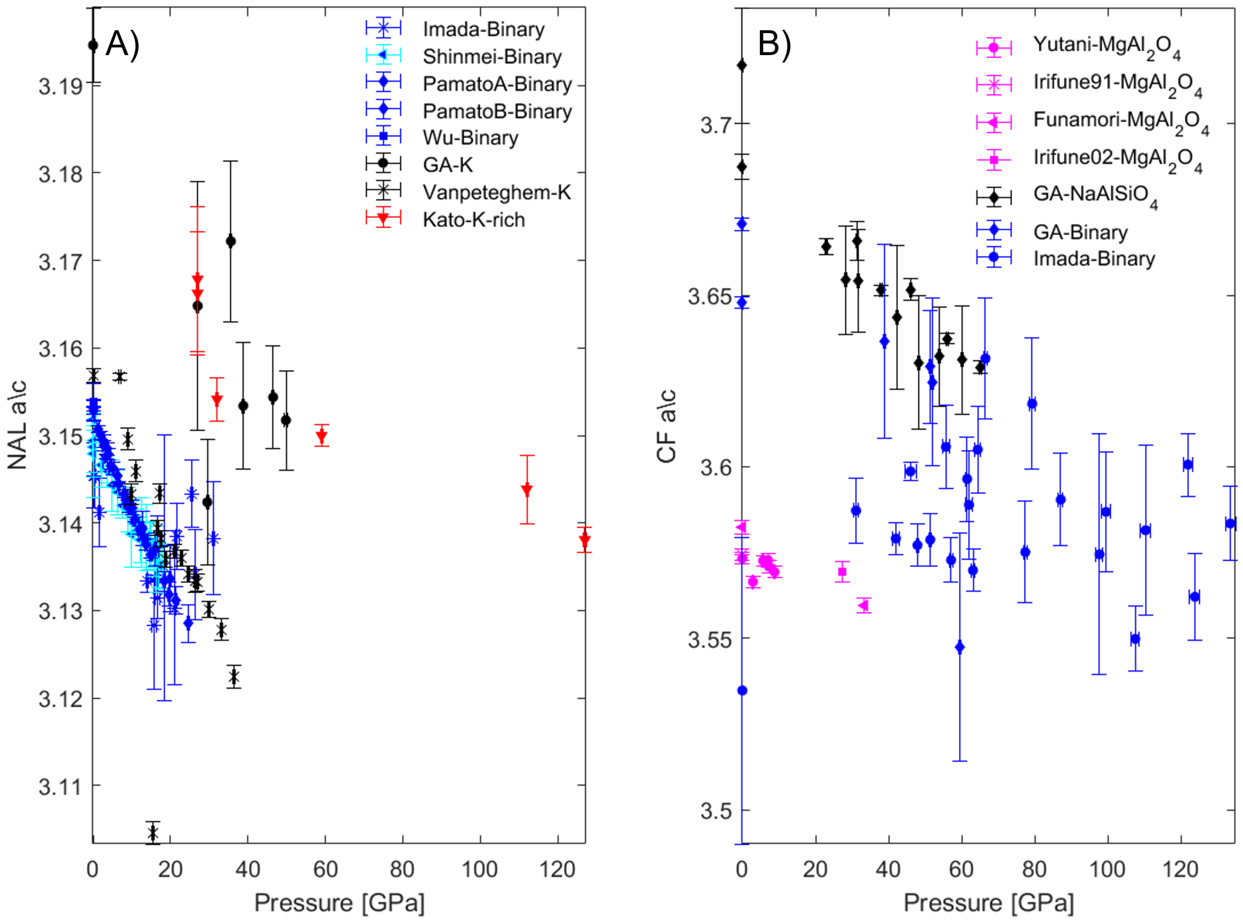

Figure S6: Axial ratios (a/c) of A) NAL and B) CF. 'Binary' indicates that the phase composition falls somewhere on the nepheline-spinel binary; 'K' indicates that the phase contains small amounts of potassium, with 'K-rich' indicating 10 mole% or more of kalsilite. References: Irifune et al. (1991); Yutani et al. (1997); Funamori et al. (1998); Irifune et al. (2002); Vanpeteghem et al. (2003); GA: Guignot and Andrault (2004); Shinmei et al. (2005); Imada et al. (2012); Kato et al. (2013); Pamato et al. (2014); Pamato (2014); Wu et al. (2016).

References

Armstrong L, Skora S, Walter MJ (2012) Mineralogy of subducted clay and clay restite in the lower mantle. In: AGU Fall Meeting Abstracts, pp DI41A–2286

Funamori N, Jeanloz R, Nguyen JH, et al. (1998) High-pressure transformations in $MgAl_2O_4$. Journal of Geophysical Research: Solid Earth 103(B9):20813–20818. https://doi.org/10.1029/98JB01575

Gale A, Dalton CA, Langmuir CH, et al. (2013) The mean composition of ocean ridge basalts. Geochemistry, Geophysics, Geosystems 14(3):489–518. https://doi.org/10.1029/2012GC004334

Guignot N, Andrault D (2004) Equations of state of Na-K-Al host phases and implications for MORB density in the lower mantle. Physics of the Earth and Planetary Interiors 143:107–128. https://doi.org/10.1016/j.pepi.2003.09.014

Imada S, Hirose K, Komabayashi T, et al. (2012) Compression of $Na_{0.4}Mg_{0.6}Al_{1.6}Si_{0.4}O_4$ NAL and Ca-ferrite-type phases. Physics and Chemistry of Minerals 39(7):525–530. https://doi.org/10.1007/s00269-012-0508-x

Irifune T, Fujino K, Ohtani E (1991) A new high-pressure form of $MgAl_2O_4$. Nature 349(6308):409–411. https://doi.org/10.1038/349409a0

Irifune T, Naka H, Sanehira T, et al. (2002) In situ X-ray observations of phase transitions in $MgAl_2O_4$ spinel to 40 GPa using multianvil apparatus with sintered diamond anvils. Physics and Chemistry of Minerals 29(10):645–654. https://doi.org/10.1007/s00269-002-0275-1

Ishii T, Kojitani H, Akaogi M (2012) High-pressure phase transitions and subduction behavior of continental crust at pressure–temperature conditions up to the upper part of the lower mantle. Earth and Planetary Science Letters 357:31–41. https://doi.org/10.1016/j.epsl.2012.09.019

Ishii T, Miyajima N, Criniti G, et al. (2022) High pressure-temperature phase relations of basaltic crust up to mid-mantle conditions. Earth and Planetary Science Letters 584:117472. https://doi.org/10.1016/j.epsl.2022.117472

Kato C, Hirose K, Komabayashi T, et al. (2013) NAL phase in K-rich portions of the lower mantle. Geophysical Research Letters 40(19):5085–5088. https://doi.org/10.1002/grl.50966

Pamato MG (2014) Single-crystal elasticity of Al-rich phases in the Earth's transition zone and lower mantle. Disseration, Universität Bayreuth (Germany)

Pamato MG, Kurnosov A, Boffa Ballaran T, et al. (2014) Hexagonal $Na_{0.41}[Na_{0.125}Mg_{0.79}Al_{0.085}]_2[Al_{0.79}Si_{0.21}]_6O_{12}$ (NAL phase): Crystal structure refinement and elasticity. American Mineralogist 99(8-9):1562–1569. https://doi.org/10.2138/am.2014.4755

Plank T, Langmuir CH (1998) The chemical composition of subducting sediment and its consequences for the crust and mantle. Chemical geology 145(3-4):325–394. https://doi.org/10.1016/S0009-2541(97)00150-2

Ricolleau A, Perrillat Jp, Fiquet G, et al. (2010) Phase relations and equation of state of a natural MORB: Implications for the density profile of subducted oceanic crust in the Earth's lower mantle. Journal of Geophysical Research: Solid Earth 115(B8). https://doi.org/10.1029/2009JB006709

Shinmei T, Sanehira T, Yamazaki D, et al. (2005) High-temperature and high-pressure equation of state for the hexagonal phase in the system $NaAlSiO_4$ - $MgAl_2O_4$. Physics and Chemistry of Minerals 32(8):594–602. https://doi.org/10.1007/s00269-005-0029-y

Vanpeteghem CB, Ohtani E, Litasov K, et al. (2003) The compressibility of hexagonal Al-rich NAL phase: similarities and differences with calcium ferrite-type (CF) phase with implications for the lower mantle. Physics of the Earth and Planetary Interiors 138(3-4):223–230. https://doi.org/10.1016/S0031-9201(03)00155-9

Wu Y, Yang J, Wu X, et al. (2016) Elasticity of single-crystal NAL phase at high pressure: A potential source of the seismic anisotropy in the lower mantle. Journal of Geophysical Research: Solid Earth 121(8):5696–5707. https://doi.org/10.1002/2016JB013136

Yutani M, Yagi T, Yusa H, et al. (1997) Compressibility of calcium ferrite-type $MgAl_2O_4$. Physics and Chemistry of Minerals 24(5):340–344. https://doi.org/10.1007/s002690050047
